# Supplementary material for: Circulating tumor cells shed large extracellular vesicles in capillary bifurcations that activate endothelial and immune cells
Source: bioRxiv. 2024 Dec 18:2024.04.17.589880. Originally published 2024 Apr 20. Preprint. [Version 2] doi: 10.1101/2024.04.17.589880 (PMC11042361; doi:10.1101/2024.04.17.589880)
Supplement: Supplement 1 [file media-1.pdf]

Supplementary Information for

Title: Circulating tumor cells shed large extracellular vesicles in capillary-sized bifurcations

Authors: Angelos Vrynas[1], Sara Arfan[2], Karishma Satia[3,4], Salime Bazban-Shotorbani[1], Mymuna Ashna[1], Aoyu Zhang[1], Diana Visan[1], Aisher Chen[1], Mathew Carter[3,4,5], Fiona Blackhall[4,5,6], Kathryn L. Simpson[3,4,7], Caroline Dive[3,4,7], Paul Huang[2,8], Sam H. Au[1,8,*]

* Corresponding author: s.au@imperial.ac.uk

The file includes:

Supplementary Materials and Methods

Supplementary Figures S1 to S22

Supplementary Tables S1 to S7

Supplementary Movies S1 to S7

Supplementary Materials & Methods:

Chemical treatment of cells

Single cell and/or cluster suspensions of MDA-MB 231 cells were pre-treated with Cytochalasin D (Cyto-D) (Merck Life Science, UK), or Colchicine (Colch) (Merck Life Science, UK), for 3 hr at 37°C/5% CO_2, at final working concentrations of 10 µM, 50 µM respectively, as required.

For internalization inhibition studies, HUVECs, THP-1 monocytes or THP-1 derived M1 macrophages were pre-treated with Cyto-D or 5-(N-Ethyl-N-isopropyl)-Amiloride (EIPA) for 3hr at 37°C/5% CO_2, at final working concentrations of 10 µM and 50 µM, respectively.

To generate positive controls of disrupted HUVEC endothelium, HUVECs were pre-treated with tumor necrosis factor alpha (TNF-α) at a final working concentration of 0.1 µg/ml for 30 hr.

To evaluate intracellular production of nitric oxide by HUVECs, untreated or LEV co-cultured HUVECs (elaborated later) were stained with DAF-FM dye (ThermoFischer Scientific) for 1 hr at a final concentration of 5µM at 37°C/5% CO_2.

Viability assay

100 µl of cell suspensions (10^5 cells/ml) were added in each well of a 96-well plate and left to attach and grow for a day. Next, cells were treated with Cyto-D or Colch, as described above, for either 3 or 24 hr at 37°C/5% CO_2. Viability of cells at 0 hr was also examined as a control. The media was removed at the relevant timeframe and the staining dye for the live/dead assay was added for 15 mins at 37°C/5% CO_2. A staining solution consisting of 1X Propidium Iodide (Thermofischer Scientific, UK), 5 µM Calcein-AM (5 µM) and 16.2 µM Hoechst 33342 in 1 ml media was added to cells. For each condition, 4-5 multifluorescent images were obtained at 20x magnification using the Nikon microscope. Alive cells were defined as Calcein$^+$, Hoechst$^+$ and Propidium Iodide$^-$, whereas dead cells as Calcein$^-$, Hoechst$^+$ and Propidium Iodide$^+$. Viability was assessed by extrapolating the percentage of alive cells compared to overall number of cells (Hoechst-positive). Images were analyzed using the Nikon software.

Post transit cell analysis

50 µl of suspensions of cells and their shed LEVs were collected into wells of 96 well plates as described above. The diameters of LEVs (Hoechst 33542-negative, Calcein-AM positive) were measured using NIS-Elements. Cell death post-transit was assessed by performing a viability assay as elaborated above 3 hours after transit and calculating the percentage of dead cells per condition (n=3). LEVs and cells per condition were enumerated manually (n=3), by analyzing multiple representative multifluorescent images across each well, establishing thus a LEV : cell number ratio.

Scanning electron microscopy

Cells and their shed LEVs were collected as described above, and the solution was centrifuged at 180 x g for 5 min to separate cells (pellet) and retain LEVs in the supernatant (described below in more detail). LEVs were transferred onto 10 x 10 mm square glass slides that were positioned into wells of a 24 well plate. After 1 day, the media was removed and the glass slides were fixed with 4% formaldehyde (diluted in PBS) for 15 min at room temperature to crosslink proteins. Then, glass slides were chemically treated with 1% (v/v) Osmium tetroxide (Agar Scientific, UK) to crosslink lipids. The fixed samples were dehydrated with a sequence of washes with increasing concentrations of ethanol, starting from 30% and continuing with 40%, 50%, 60%, 70%, 80%, 90% and 100% (v/v) ethanol. After dehydration, the samples were chemically dried, by using Hexamethyldisilazane (Merck, UK) for 3 mins. A sticky carbon tape was attached to each square glass slide and silver-containing glue was brushed onto the samples to enhance conductivity. Finally, a 15 µm thick layer of Nickel was deposited onto the samples using a sample sputter coater (Q150TS, Quorum technologies, UK), to improve image resolution. Prepared samples were loaded into the stage holder of the scanning electron microscope (Zeiss Auriga 40 Cross beam scanning electron microscope, Zeiss, Germany). 5 kV of acceleration voltage (using secondary electron detection) was applied to the sample. Samples were scanned to identify LEVs and images at 1000-8000x magnification.

Post transit cell proliferation

To evaluate the proliferative capabilities of MDA-MB231 cells after transit, 5 x 10^5 cells were flowed through a microfluidic device, the effluent collected, and cells separated from LEVs as

described above. Cells were enumerated using a hemocytometer (as above). 250 µl of cell suspension of 0.5×10^6 cells/ml density were added to wells of a 48-well plate. Equal numbers of cells that did not transit through devices were added to separate wells as controls. Images were taken 4 hr after seeding and then every day up to 7 days post-seeding. In other experiments, post-transit cells and control cells were left to grow for 3 days in 48 well plates and were then harvested and enumerated using a hemocytometer.

Flow cytometry

MDA-MB 231 cells, HUVECs, monocytes or purified LEVs (the latter derived from MDA-MB 231 cells as described above) were stained as described above (see also Supplementary Table 5). 500 µl of each sample (10×10^5 cells/ml or 20×10^4 LEVs/ml) were loaded into an Amnis Cell Stream flow cytometer (Luminex, US) and samples were run at a flow rate of 20 µl/min. At least 1000 events per sample and condition were recorded. Excitation and emission wavelengths used for obtaining relevant dot plots are depicted in Supplementary table 6. For all histograms generated, antibody fluorescence intensity was represented in the x axis and frequency in the y axis. As a general approach, unstained samples and single-color controls were used for setting gating thresholds.

For LEVs internalization studies, pre-stained cells with a red cell tracker (HUVECs or monocytes or M1 macrophages) were co-cultured independently with pre-stained LEVs with green cell tracker for 16 hr. Internalization frequency was defined as the number of double positive events, cells that had internalized LEVs compared to overall number of cells. Where required, cells were pre-treated with Cyto-D, EIPA or combination, as described elsewhere and then LEVs were added. In each case, unstained cells, untreated cells (stained) and LEVs alone (stained), were used as controls for gating the limits for double positive dot plots (cells that had internalized LEVs) and samples were analyzed as above. For relevant experiments, fluorescent beads of 5- and 10 µm (microParticles GmbH, Germany) were used.

Protein concentration & gel electrophoresis

Initially, suspensions of each fraction were washed 1x with chilled (4°C) PBS and lysed using 300 µl of radioimmunoprecipitation assay (RIPA) lysis buffer (Thermo Fischer Scientific, UK) for 15 min on ice in a plate shaker. All fractions were centrifuged at 18900 x g for 15 min to

remove debris and the supernatant containing the lysate was collected. All other previous centrifugation steps were at 180 x g for 5 min and at 9600 x g for 30 min, for cells and LMPs, respectively. The protein lysates were stored at -80°C or placed on ice for immediate use.

Protein quantity of MDA-MB 231 control cells, cells post-transit (bifurcated capillary devices) and LEVs were measured using Bicinchoninic acid (BCA) assay kit (Life Technologies, UK), per manufacturer's instructions. The absorbance of each sample at 562 nm was analyzed using a Varioskan Flash plate reader (ThermoFischer Scientific, UK).

For qualitative protein analysis, gel electrophoresis was performed. The separating gel was prepared by mixing 1.6 ml PBS, 2 ml Acrylamide/Bis-acrylamide 30% (v/v) solution, 1.3 ml 1.5 M Tris (Life Technologies, UK), 50 µl 10% (v/v) Sodium Dodecyl Sulfate (Thermo Fischer Scientific), 50 µl 10% (v/v) Ammonium Persulfate (Bio Rad, UK) and 2 µl tetramethylethylenediamine (Bio Rad, UK). The stacking gel was prepared by mixing 1.4 ml PBS, 0.33 ml Acrylamide/Bis-acrylamide 30% (v/v) solution, 0.25 ml 1.5 M Tris, 20 µl 10% (v/v) Sodium Dodecyl Sulfate, 20 µl 10% (v/v) Ammonium Persulfate and 2 µl tetramethylethylenediamine. Both gels were left to polymerize at room temperature for 20 min. Both the separating and stacking gel were enclosed in a glass cassette (Bio Rad, UK) and incorporated in the electrophoresis chamber (Bio Rad, UK). 250 µg/ml of protein samples (15 µl) are mixed with 5µl Laemmli protein buffer (Bio Rad, UK) and heated for 10 min at 95°C. 12 µl of each sample and protein ladder solution (Bio Rad, UK) are added across the wells of the gel. The electrophoresis chamber was filled with 10% (v/v) Sodium Dodecyl Sulfate and the samples were run for 120 min at 125 V and 400 A. The gel was then removed carefully from the enclosed glass cassette and stained with 50 ml Coomassie blue (Bio Rad) in a glass sealed container for 1 hr at room temperature on a plate shaker (Thermo Fischer Scientific, UK). The staining solution was removed and the gel was destained using 50 ml of Coomassie brilliant blue R-250 destaining solution (Bio rad, UK) for 1 hr at room temperature on a plate shaker. Images were obtained with a mobile phone (Redmi, China).

HUVEC permeability assays

$15x10^3$ HUVECs were cocultured in wells of a 96 well plate with $5x10^3$ purified LEVs derived from MDA-MB 231 cells as described above. Equal numbers of HUVES but cultured without LEVs were used as negative controls. HUVECs were pre-stained with a CMFDA green cell

tracker. At 0hr, both fluorescent and brightfield images were obtained at 10x and 20x magnification. After 30hr, similar images were obtained again to characterise the extent of HUVEC monolayer disruption. In other experiments, HUVECs were stained with VE-cad and Hoechst instead of CDFMA cell tracker as described above. Cell-free vs. cell-containing areas were extrapolated using ImageJ.

HUVEC monolayer disruption was further characterized by performing a dextran permeability assay. Millicell hanging cell culture inserts of either 0.4 µm or 8 µm pores (Merck, UK) were transferred in wells of a 24-well plate. The well was filled with 900 µl around the cell culture insert and 200 µl of HUVECs ($75x10^3$ cells/ml) were then transferred inside the cell culture inserts and left for a day to attach and form the monolayer. Then, HUVECs were treated with $5x10^3$ purified LEVs derived from MDA-MB 231 cells (as described elsewhere) for 30 hr. Then, 100 µl of fresh media was added to 0.4 µm or 8 µm, containing either 10,000 molecular weight Dextran Alexa Fluor™ 647 (ThermoFischer Scientific, UK) or $10x10^4$ cells/ml pre-stained MDA-MB 231 cells (green cell tracker), respectively, for 24 hr. 100 µl of media was collected from the bottom of the well at 24 hr and fluorescence was measured using a Varioskan Flash plate reader (ThermoFischer Scientific, UK) at 650/668 nm values of peak excitation/emission. The fluorescence values obtained at 24hr were normalized initially based on blank samples (cell-free media) and baseline subtracted using fluorescence at 0 hr.

Monocyte differentiation

CM was collected from untreated HUVECs or HUVECs cocultured with LEVs (derived from MDA-MB 231 cells as above), as described above. $15x10^3$ THP-1 monocytes were transferred into wells of a 96-well plate and 100% of 200 µl of CM from above conditions was added for 30 hr. Additional experimental conditions included the addition of either $5x10^3$ LEVs or CM collected from LEVs on monocytes for 30 hr. Untreated monocytes were used as a negative control. After 30hr, media was removed and monocytes were stained via immunocytochemistry for CD206 and TNF-a, as described above. Multifluorescent and brightfield images were obtained at 20x magnification, using the confocal microscope. Alternatively, $10x10^4$ THP-1 monocytes were added in wells of a 24-well plate and all above experimental conditions were repeated. 500 µl from each sample was collected and analyzed

via flow cytometry as described above. The frequency of CD206+ events was calculated to estimate the differentiation percentage of monocytes to M2 macrophages.

Monocyte adhesion

$15x10^3$ monocytes were stained with a CMTPX red Cell Tracker and were then transferred in wells of a 96 well plate. Then, monocytes were treated independently with $5x10^3$ purified LEVs (derived from MDA-MB 231 cells as above), with 100% CM collected from LEVs and 100% CM collected from MDA-MB 231 cells (as described above) for 30 hr. Untreated monocytes were used as a negative control. Fluorescent images of each well were obtained before and after media removal (removing non-adhered monocytes) and the percentage of remaining adhered cells was calculated.

Alternatively, $15x10^3$ HUVECs were added in wells of a 96-well plate and left to grow for 1 day. In the meantime, $30x10^3$ THP-1 monocytes were cultured in wells of a 48-well plate, left to grow for 1 day and then stained with a CMFDA green cell tracker. HUVECs or THP-1 monocytes were treated with $5x10^3$ or $10x10^3$ purified LEVs (derived from MDA-MB 231 cells as above) for 30hr, respectively. Negative control of both untreated HUVECs and untreated THP-1 monocytes were used. After 30hr, media was removed both from HUVECs and monocytes to remove non-internalized LEVs and four experimental conditions were performed: a) untreated THP-1 monocytes were added to untreated HUVECs monolayers, b) untreated THP-1 monocytes were added to LEV-pre-treated HUVECs monolayers, c) LEV-pre-treated THP-1 monocytes were added to untreated HUVECs monolayers, and d) LEV-pre-treated monocytes were added to LEV-pre-treated HUVECs monolayers. Using a Varioskan flash plate reader, the green fluorescence of monocytes at 0 hr was measured at 492/517 nm values of peak excitation/emission. After 4hrs of co-culture, the media was removed from each well and fresh media was added. Fluorescence of monocytes was measured by plate reader as described above. Cell-free media was used for normalization and the percentage of remaining fluorescence after media removal (removing non-adhered monocytes) was estimated.

Monocyte proliferation assay

$12x10^3$ monocytes were added in each well of a 96-well plate and left to grow for 1 day. Then, they were treated independently with $4x10^3$ purified LEVs (derived from MDA-MB 231 cells as above) or 100% CM collected from LEVs (as described above) for altogether 48 hrs. Untreated monocytes were used as a negative control. To evaluate monocyte proliferation the MTT cell proliferation assay kit (Abcam, UK) was used, per manufacturer's instructions. The absorbance at 590 nm of each well was read using a Varioskan flash plate reader and normalized as described elsewhere.

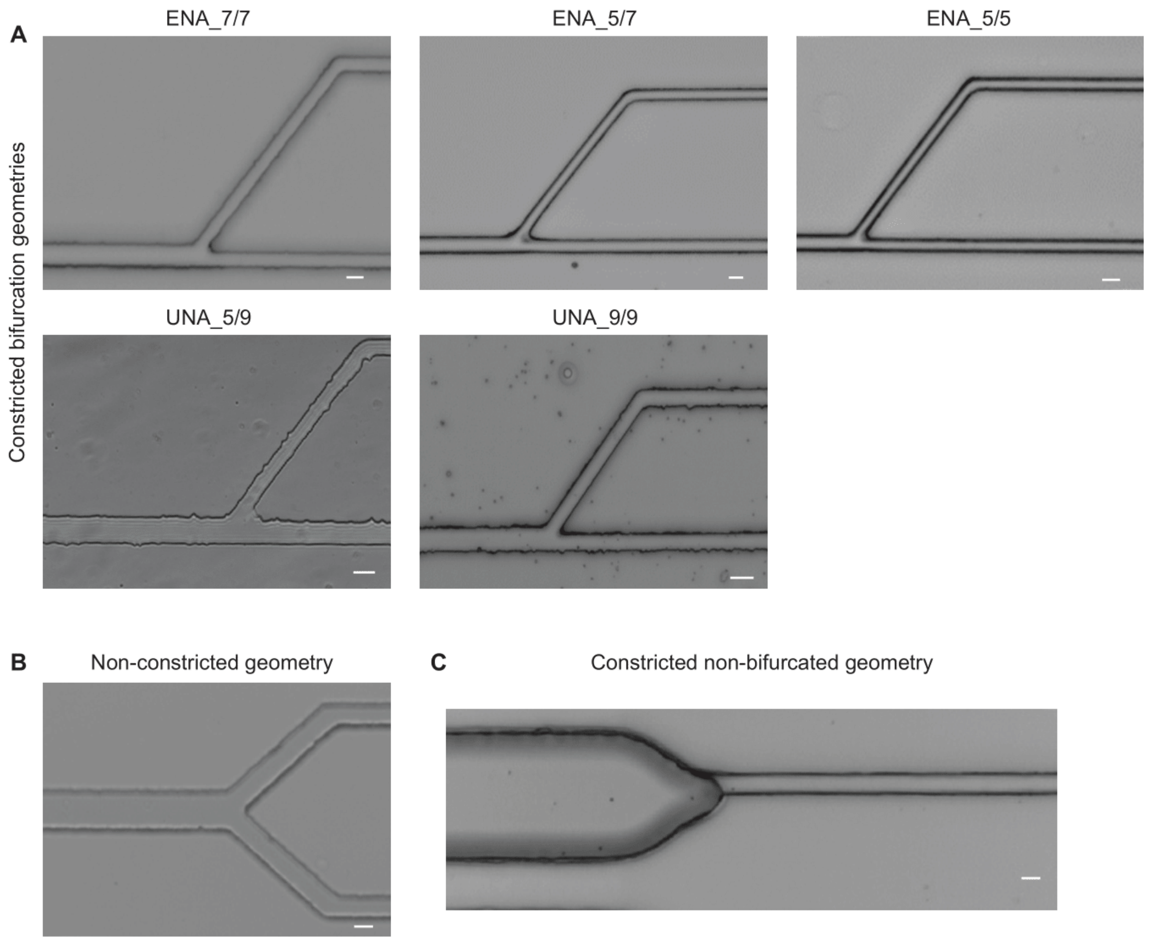

Supplementary Fig. 1. Microfluidic devices. (**A**) Brightfield images of various constricted bifurcation variants. Scale bar: 10 µm (excluding for UNA_9/9: Scale bar: 15 µm). (B) Brightfield image of a non-constricted geometry. Scale bar: 20 µm. (C) Brightfield image of a constricted non-bifurcated geometry. Scale bar: 7 µm.

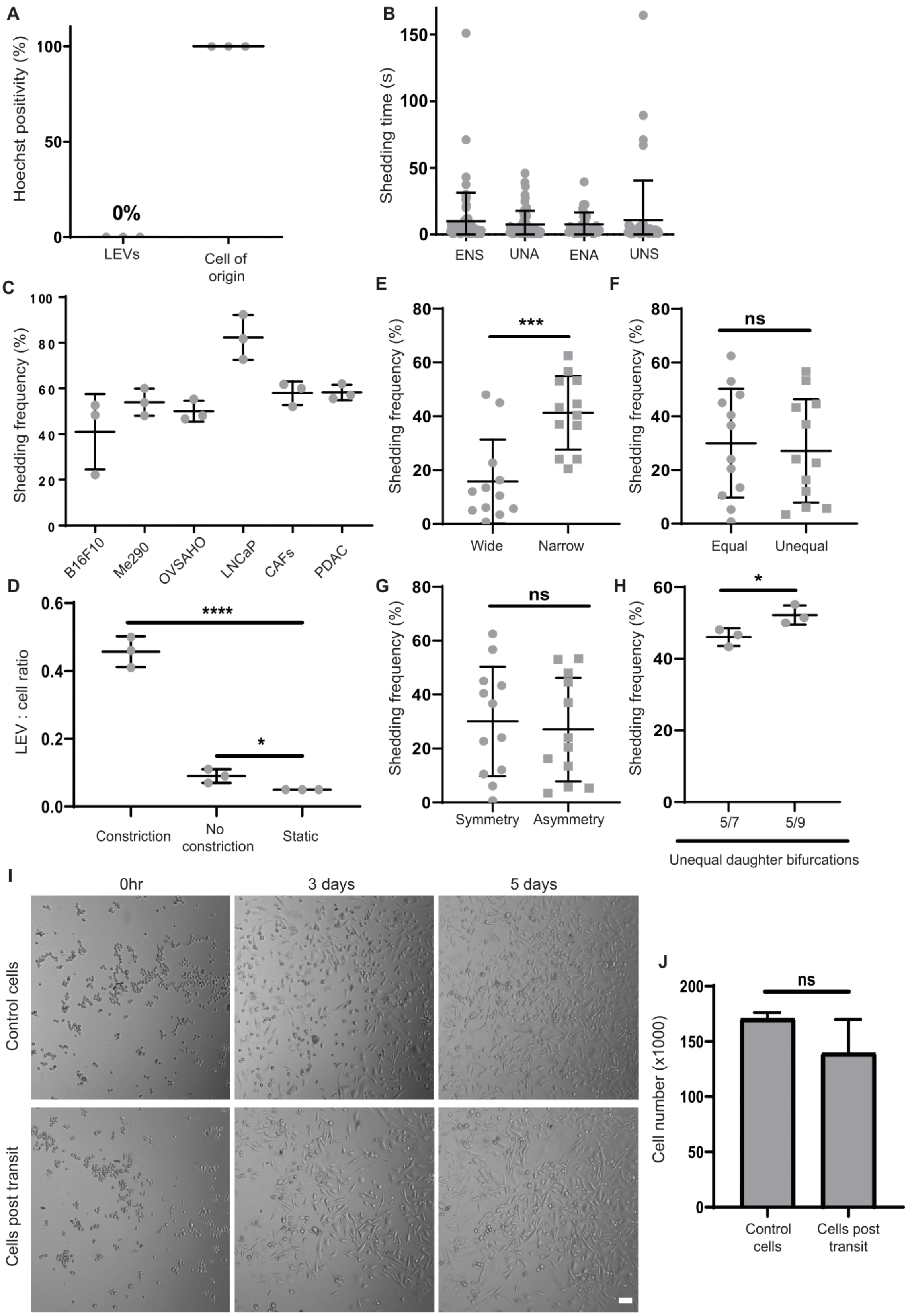
A
Hoechst positivity (%)
0%
LEVs
Cell of origin
B
Shedding time (s)
ENS
UNA
ENA
UNS
C
Shedding frequency (%)
B16F10
Me290
OVSAHO
LNCaP
CAFs
PDAC
D
LEV : cell ratio
****
*
Constriction
No constriction
Static
E
Shedding frequency (%)
***
Wide
Narrow
F
Shedding frequency (%)
ns
Equal
Unequal
G
Shedding frequency (%)
ns
Symmetry
Asymmetry
H
Shedding frequency (%)
*
5/7
5/9
Unequal daughter bifurcations
I
0hr
3 days
5 days
Control cells
Cells post transit
J
Cell number (x1000)
ns
Control cells
Cells post transit

Supplementary Fig. 2. Biomechanical investigation of LEV biogenesis. (**A**) Percentage of Hoechst-positive MDA-MB 231 cells or their derived LEVs after transit through bifurcation variant ENA using live imaging (n=3). (B) MDA-MB 231 cells' shedding time in 4 different capillary bifurcation variants (n=66 for ENS, n=61 for UNA, n=31 for ENA, n=46 for UNS). Each data point represents a single cell. (C) Percentage of shedding frequency for melanoma (B16F10, Me290), ovarian (OVSAHO), prostate (LNCaP), pancreatic (PDAC) cancer cell lines and primary cancer-associated fibroblasts during live imaging (n=3). (D) LEV : cell ratio, enumerated post MDA-MB 231 cells transit in bifurcation variant UNA_5/9 or non-constricted geometry or static conditions (n=3). (E-H) Percentage of MDA-MB 231 cells shedding frequency in different bifurcation variants (E) wide vs narrow (n=12) (F) equal vs unequal (n=12) (G) symmetry vs asymmetry (n=12) and (H) unequal capillary bifurcation geometries UNA_5/7 vs UNA_5/9 during live imaging (n=3). (I) Brightfield images of control cells or cells post transit (MDA-MB 231 cells) at 0hr, 3 days and 5 days. Scale bar: 100 µm. (J) Quantification of MDA-MB 231 cell number 72 hrs post transit through bifurcation variant ENA versus control non-transiting cells.

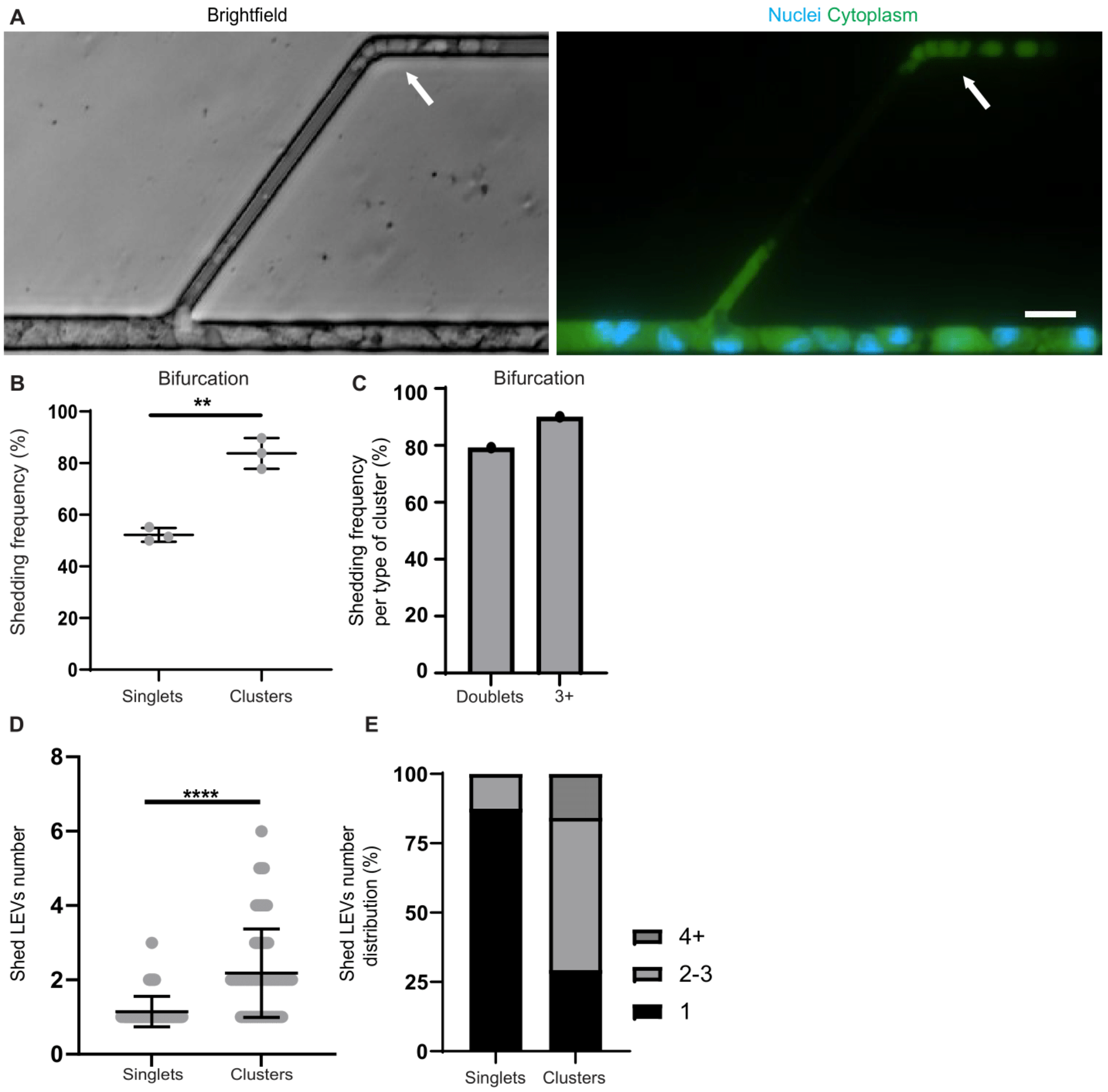

Supplementary Fig. 3. Investigation of LEV biogenesis from tumor cell clusters. (**A**) Brightfield & multifluorescent image of trapped large cluster of MDA-MB231 cells shedding multiple LEVs (arrow) in bifurcation variant UNA_5/9. Cytoplasm was stained with CMFDA cell tracker (green) and nuclei with Hoechst-33342 (blue). Arrow depicts the multiple shed LEVs. Scale bar: 20 µm. (B) Percentage of either single MDA-MB 231 cells or clusters that shed at least once during live imaging in bifurcation variant UNA_5/9. (n=3). (C) Percentage of MDA-MB 231 cell clusters that shed per type of cluster, 2-cell cluster (doublet) (n=101) or 3-cell cluster and higher (n=40). Data were pooled together from cell transit in bifurcation variants UNA_5/9 and UNA_7/7. (D) Number of LEVs shed per single MDAMB 231 cell (n=48 singlets) or cluster (n=66 clusters) in bifurcation variant UNA_5/9. (E) Distribution of (D) per number of shed LEVs.

Supplementary Figure 4

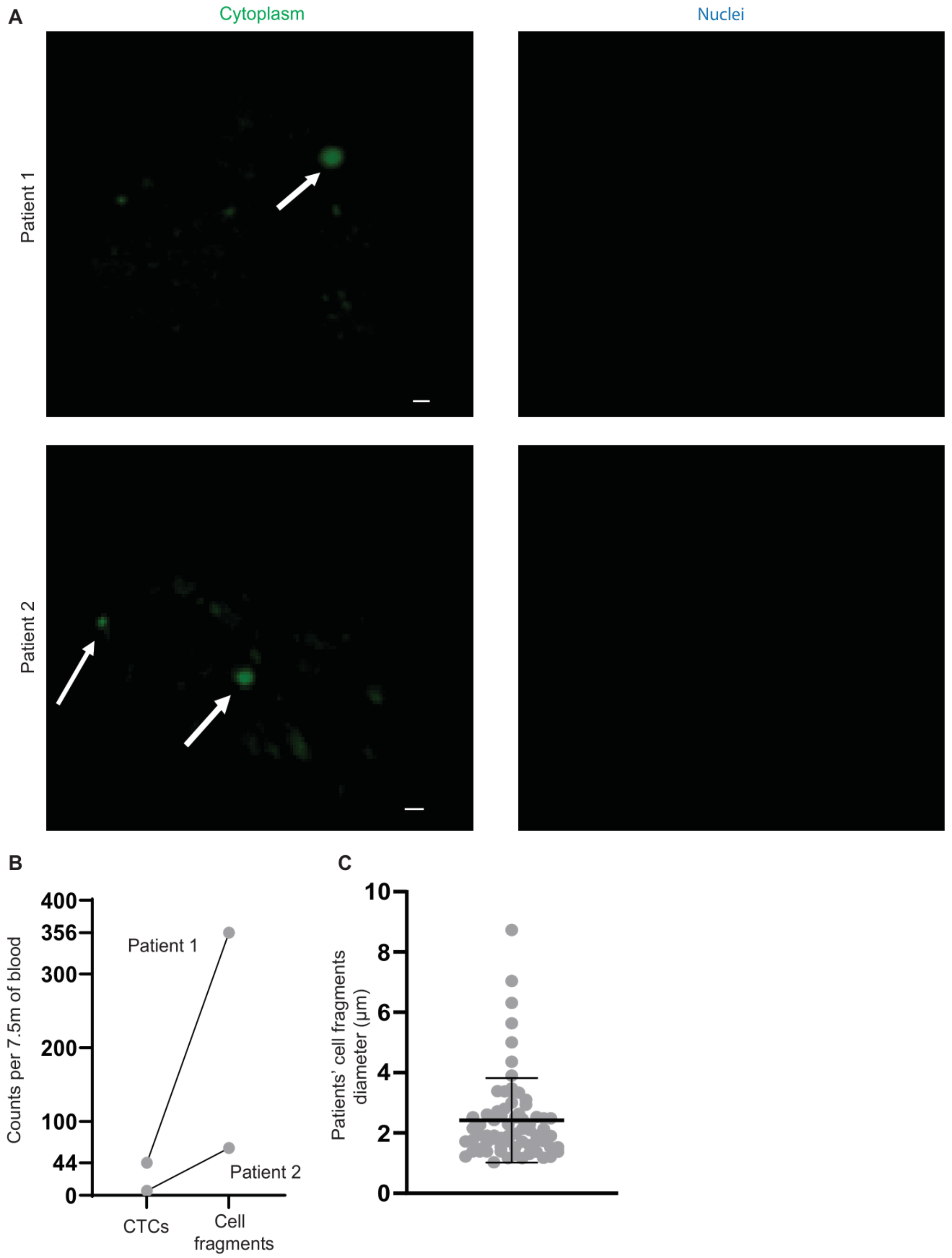

Supplementary Fig. 4. Isolation of patient cellular fragments. (**A**) Multifluorescent images of isolated cell fragments from patients with small cell lung carcinoma (patient 1: top & patient 2: bottom), stained with CMFDA for cytoplasm (green) and Hoechst-33342 for nuclei (blue) (n=2 patients). Arrows indicate cellular fragments. Scale bar: 5 µm (Patient 1) & Scale bar: 4 µm (Patient 2). (B) CTCs and cell fragments numbers from patient blood (n=2 patients). (C) Diameter (µm) of patient-isolated cell fragments (n=70 fragments).

Supplementary Figure 5

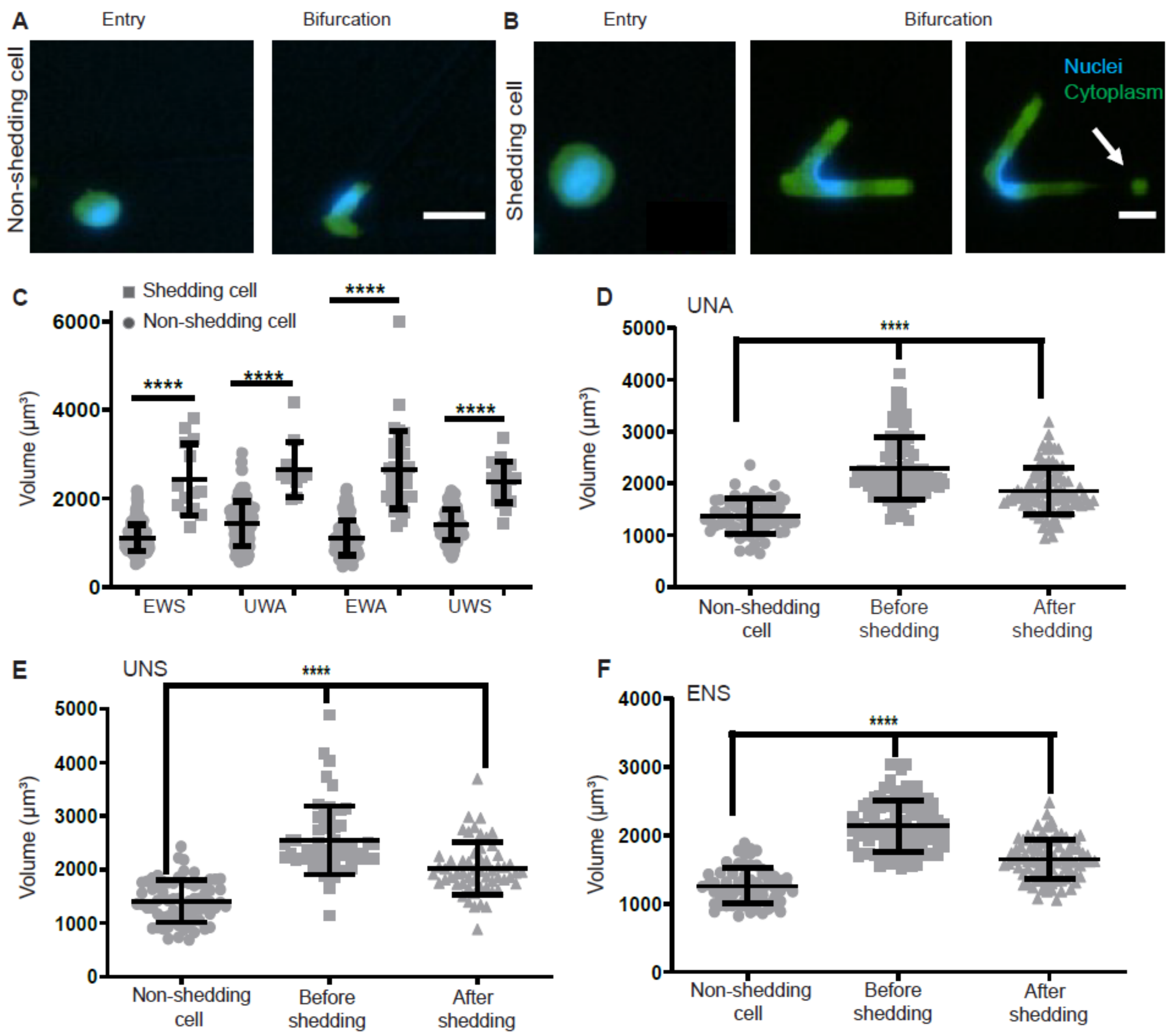

Supplementary Fig. 5. Tumor cell size promotes shedding. (**A-B**) 20x multifluorescent images of non-shedding (A) or shedding (B) (arrow) MDA-MB 231 cells. Cytoplasm was stained with Calcein-AM (green) and nuclei with Hoechst-33342 (blue). Scale bar: 20µm. (C) Cytoplasmic volume (μm^3) of non-shedding MDA-MB 231 cells and MDA-MB 231 cells that eventually shed, during transit in 4 bifurcated capillary geometries (EWS, UWA, EWA, UWS). (D) Cytoplasmic volume (μm^3) of non-shedding MDA-MB 231 cells (n=62) and MDA-MB 231 cells that eventually shed (before and after shedding) (n=97), during transit in bifurcation variant UNA. (E) Cytoplasmic volume (μm^3) of non-shedding MDA-MB 231 cells (n=71) and MDA-MB 231 cells that eventually shed (before and after shedding) (n=57), during transit in bifurcation variant UNS. (F) Cytoplasmic volume (μm^3) of non-shedding MDA-MB 231 cells (n=68) and MDA-MB 231 cells that eventually shed (before and after shedding) (n=92), during transit in bifurcation variant ENS. (C-F) Each data point represents a single cell.

Supplementary Figure 6

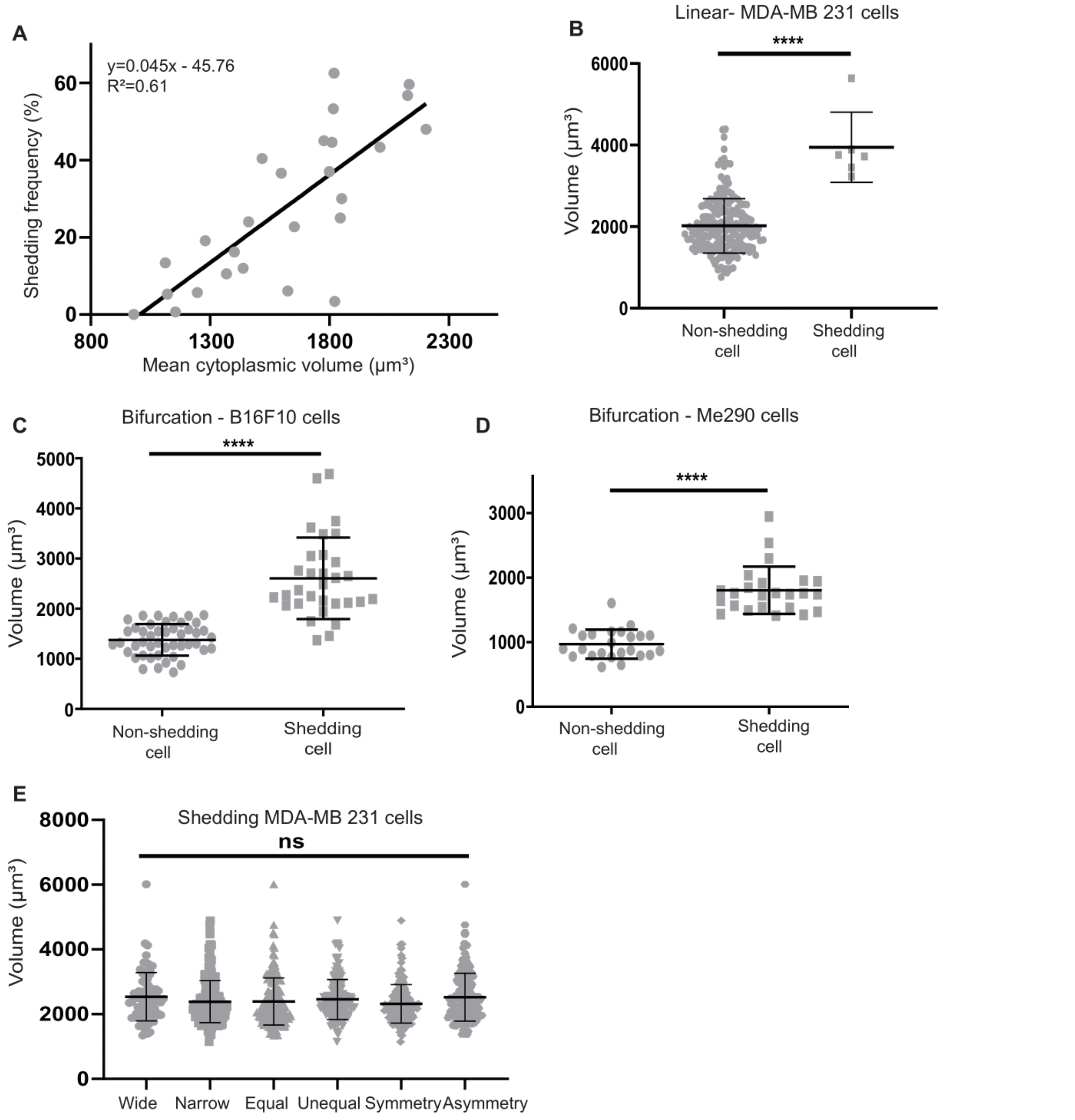

Supplementary Fig. 6. Influence of cell size on shedding during transit in various microfluidic variants. (**A**) Graph plot of shedding frequency (y axis) and mean cytoplasmic volume (μm^3) (x axis) (n=26). Data were pooled together from various bifurcation devices. (B-D) Each data point represents a single cell. (B) Cytoplasmic volume (μm^3) of non-shedding MDA-MB 231 cells (n=198) and MDA-MB 231 cells that eventually shed (n=6), during transit in constricted non-bifurcated (linear) geometry. (C-D) Cytoplasmic volume (μm^3) of non-shedding cells (n=46 for B16F10 (C), n= 24 for Me290 (D)) or shedding cells (n=31 for B16F10 (C), n= 24 for Me290 (D)), during transit in bifurcation variant UWA (C) and ENA (D). (E) Cytoplasmic volume (μm^3) of shedding MDA-MB 231 cells, per bifurcation design parameter (n=79 for wide, n=244 for narrow, n=170 for equal, n=153 for unequal, n=159 for symmetry, n=164 for asymmetry.

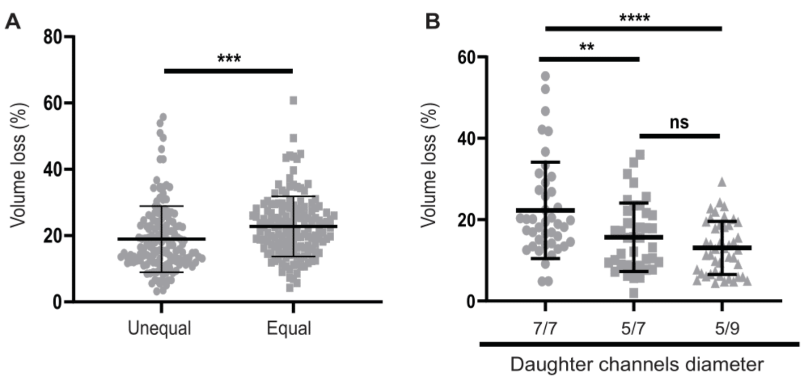

Supplementary Fig. 7. Tumor cell volume losses post shedding. (**A-B**) Each data point represents a single cell. (A) Percentage of volume loss of post shedding MDA-MB 231 cells during transit in unequal bifurcation variants (UNA, UNS) (n=154) or equal bifurcation variants (ENA, ENS) (n=148 cells). (B) Percentage of volume loss of post shedding MDA-MB 231 cells during transit in 3 independent bifurcation variants (n=42 cells for ENA_7/7, n=39 cells for UNA_5/7, n= 44 cells for UNA_5/9).

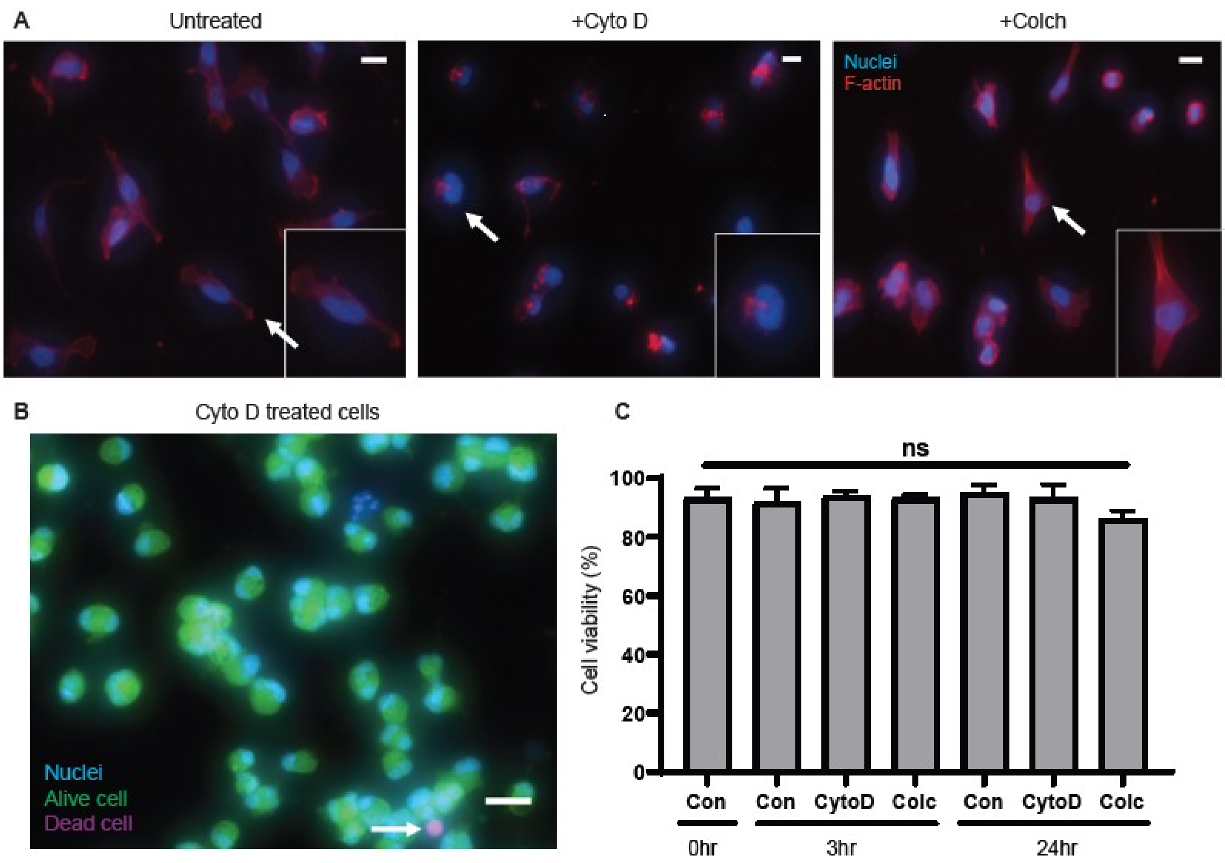

Supplementary Fig. 8. Validation of F-actin polymerization inhibitors or promoters. (**A**) Multifluorescent images of untreated (left), Cytochalasin D (Cyto-D) treated (middle), or Colchicine (Colch) treated MDA-MB 231 cells. F-actin was stained with Phalloidin (red) and nuclei with Hoechst-33342 (blue). Arrows indicate the zoomed cell (inset). Scale bar: 20 µm. (B) Multifluorescent image obtained from live/dead assay. Live cells were stained with Calcein-AM (green), dead cells with Propidium Iodide (red) and nuclei with Hoechst-33342 (blue). Arrow indicates a dead cell. Scale bar: 50µm. (C) Quantification of live/dead assay for untreated cells (Con), Cyto-D treated or Colch-treated cells at 3 or 24hrs (n=3).

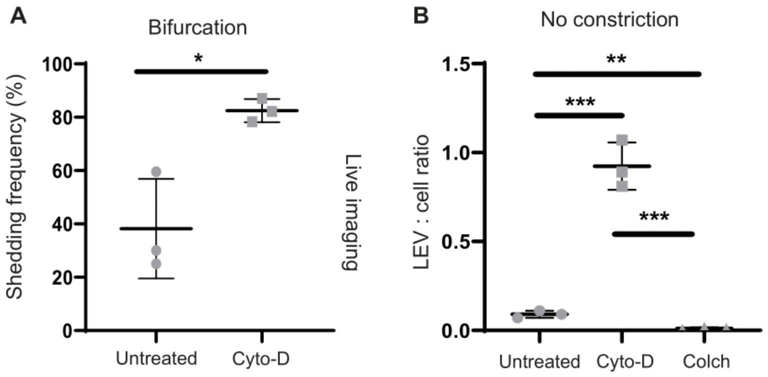

Supplementary Fig. 9. Investigation of F-actin polymerization inhibitors or promoters on single cell shedding. (**A**) Shedding frequency (%) of untreated or Cyto-D treated MDA-MB 231 cells during transit in bifurcation variant ENA (live imaging) (n=3). (B) LEV : cell ratio of untreated, Cyto-D or Colch-pre-treated MDA-MB 231 cells enumerated post transit in non-constricted geometry (n=3).

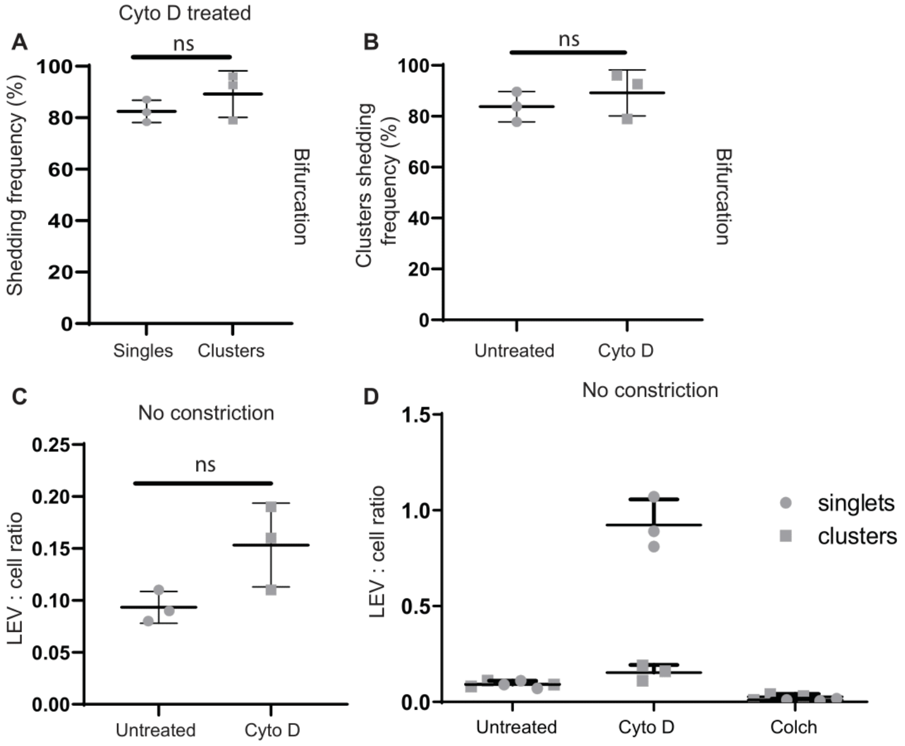

Supplementary Fig. 10. Investigation of F-actin polymerization inhibitors or promoters on clustered cells shedding. (**A**) Percentage of Cyto-D-treated clusters or Cyto-D treated single MDA-MB 231 cells that shed at least once during transit in bifurcation variant UNA_5/9 (live imaging) (n=3). (B) Percentage of Cyto-D treated clusters or untreated clusters (MDA-MB 231 cells) that shed at least once during transit in bifurcation variant UNA_5/9 (n=3). (C) LEV : cell ratio of Cyto-D treated or untreated clusters enumerated post transit in non-constricted geometry (n=3). (D) LEV : cell ratio of Cyto-D treated, Colch-treated and untreated single cells or clusters, enumerated post transit in non-constricted geometry (n=3).

Supplementary Figure 11

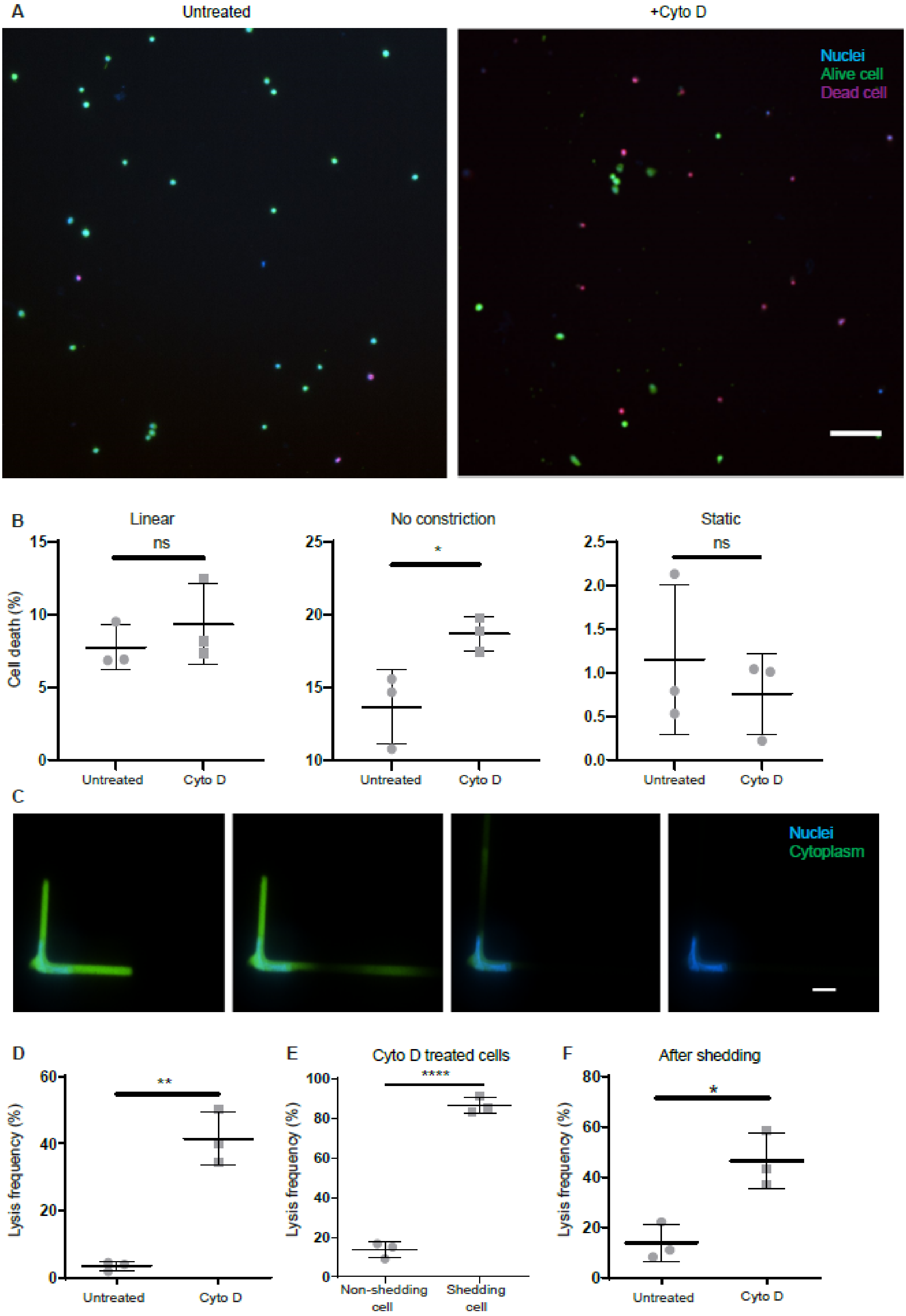

Supplementary Fig. 11. Investigation of tumor cell viability in microfluidic variants. (**A**) Multifluorescent images of a live/dead assay for untreated (left) or Cyto-D treated (right) MDA-MB 231 cells collected post transit from capillary bifurcations. Live cells were stained with Calcein-AM (green), dead cells with Propidium Iodide (red) and nuclei with Hoechst-33342 (blue). Scale bar: 100 µm. (B) Percentage of untreated or Cyto-D treated cells that were stained dead post transit in constricted non-bifurcated (linear) geometry (left), non-constricted geometry (middle) or static conditions (right) (n=3). (C) Timelapse of a MDA-MB 231 cell trapped in bifurcation variant EWA, that shed and eventually was lysed. Cytoplasm was stained with CMFDA cell tracker (green) and nuclei with Hoechst-33342 (blue). Scale bar: 10 µm. (D) Percentage of untreated or Cyto-D treated MDA-MB 231 cells that were lysed during transit in bifurcation variant ENA (n=3). (E) Percentage of non-shedding or shedding Cyto-D treated MDA-MB 231 cells that were lysed during transit in bifurcation variant ENA (n=3). (F) Percentage of untreated or Cyto-D treated MDA-MB 231 cells that were lysed post shedding, during transit in bifurcation variant ENA (n=3).

Supplementary Figure 12

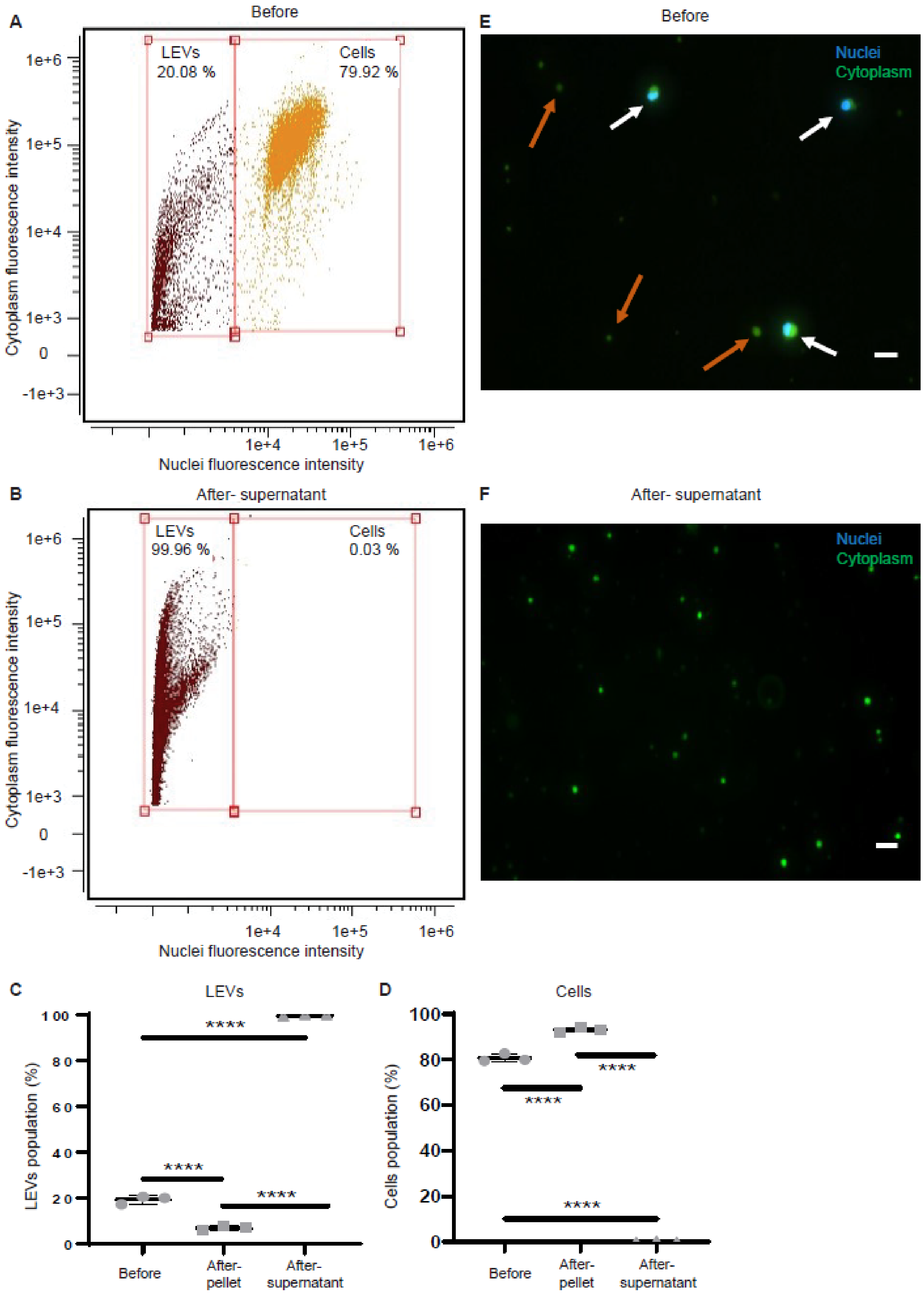

Supplementary Fig. 12. Separation of cells and their shed LEVs. (**A-B**) Dot plot of gated Cytoplasm-positive MDA-MB 231 cells and their derived cytoplasm-positive LEVs, separated based on Hoechst (nuclei) intensity, before (A) and after centrifugation (supernatant) (B). (C-D) Percentage of LEVs (C) or cells (D) that were present in the sample before centrifugation, after centrifugation (pellet) and after centrifugation (supernatant) (n=3). (E-F) 20x multifluorescent images before (E) and after centrifugation (supernatant) (F). Cytoplasm was stained with CMFDA cell tracker (green) and nuclei with Hoechst-33342 (blue). Scale bar: 20 µm. White arrows indicate cells and orange arrows indicate LEVs (E).

Supplementary Figure 13

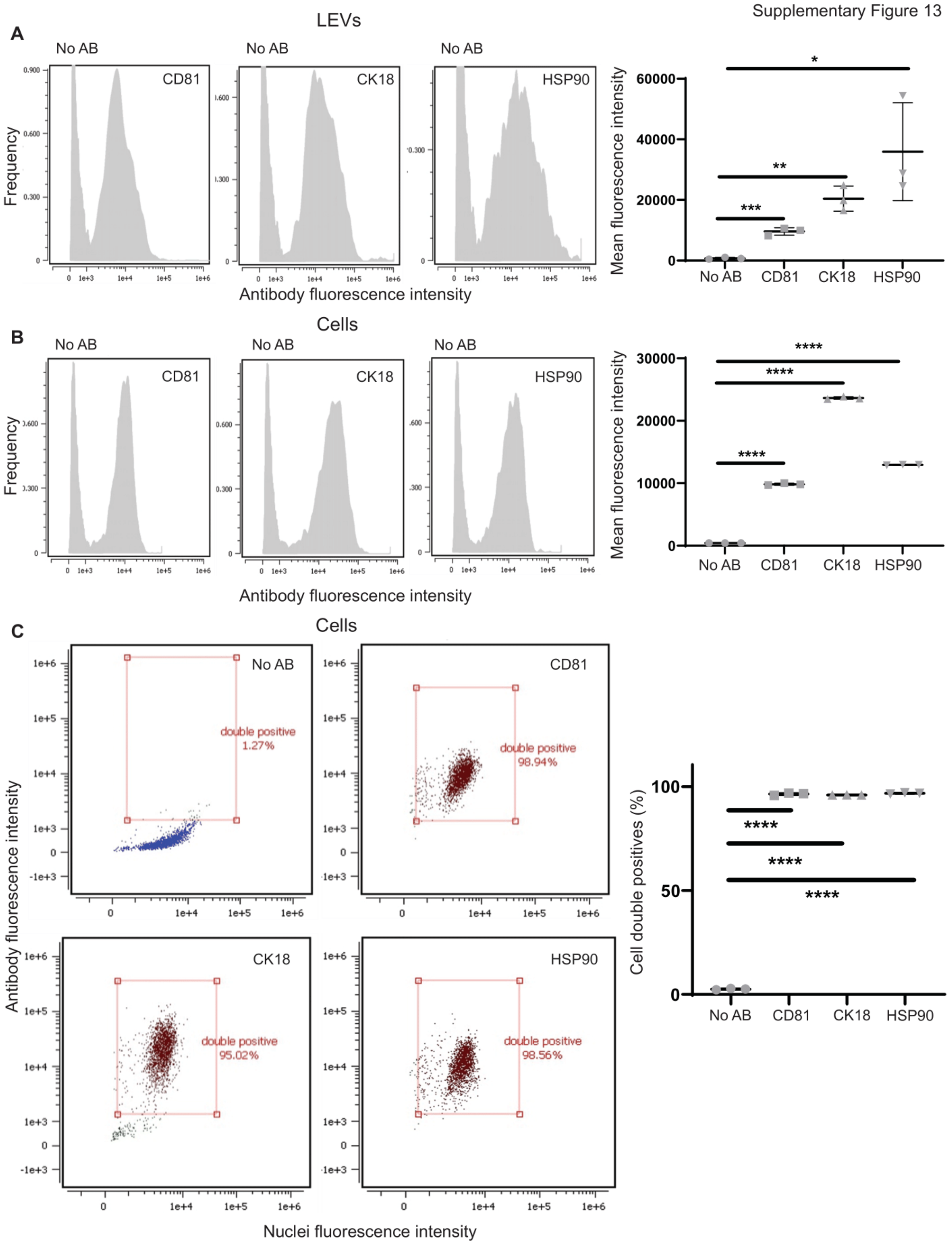

Supplementary Fig. 13. Protein inclusion on tumor cells and their shed LEVs. (**A-C**) Data obtained from flow cytometry analysis. (A) Histograms (left) & quantification (far right) of the mean fluorescence intensity of antibody-stained (Cluster of differentiation 81-CD81, Cytokeratin 18-CK18 and Heat shock protein 90-HSP90) or non-antibody stained (No AB) LEVs, derived from MDA-MB 231 cells (n=3). (B) Histograms (left) & quantification (far right) of the mean fluorescence intensity of antibody-stained (CD81, CK18 and HSP90) or non-antibody stained (No AB) MDA-MB 231 cells (n=3). (C) Dot plots (left) & quantification (far right) of the percentage of double positive events for Hoechst-positive MDA-MB 231 cells that express CD81 (top right) or CK18 (bottom left) or HSP90 (bottom right). Non-antibody stained (No AB) cells were used as control (top left) (n=3). Antibody fluorescence and Hoechst fluorescence intensity are recorded in each dot plot in the y and x axes, respectively.

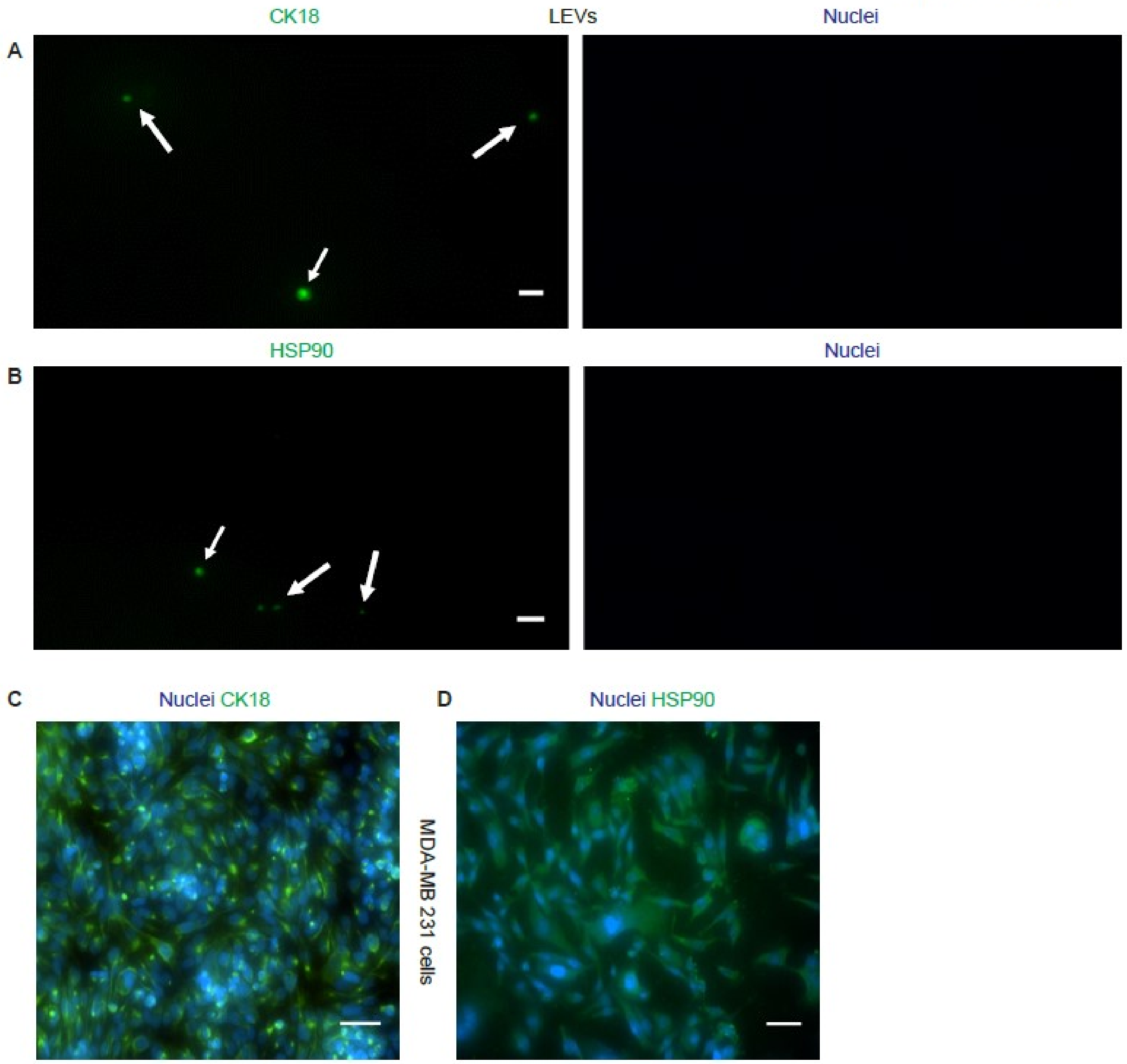

Supplementary Fig. 14. Imaging of protein expression on tumor cells and their shed LEVs. (A) Multifluorescent image of purified LEVs stained for CK18 (green) (arrows). Nuclei was stained with Hoechst-33342 (blue). Arrows indicate LEVs. Scale bar: 10 µm. (B) Multifluorescent image of purified LEVs stained for HSP90 (green) (arrows). Nuclei was stained with Hoechst-33342. Arrows indicate LEVs. Scale bar: 10 µm (C) Multifluorescent image of MDA-MB 231 cells stained for CK18 (green). Nuclei was stained with Hoechst-33342 (blue). Scale bar: 50 µm. (D) Multifluorescent image of MDA-MB 231 cells stained for HSP90 (green). Nuclei was stained with Hoechst-33342 (blue). Scale bar: 50 µm.

Supplementary Figure 15

A

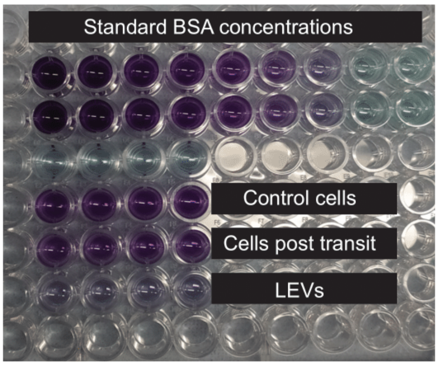

B

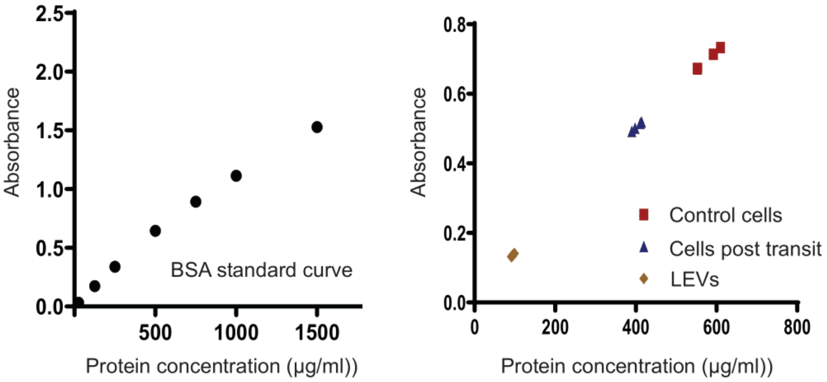

C

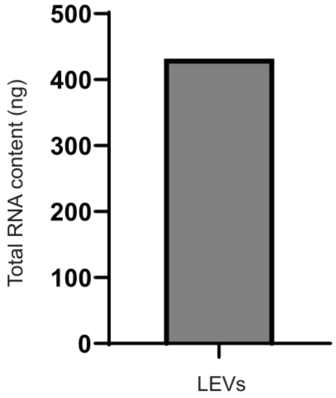

D

Gene	CT (Sample 1)	CT (Sample 2)	
IL-6	11.96	10.3	
CXCL10	10.94	8.83	
FN	11.24	6.06	
ALDH1	10.86	8.99	
BCL2	8.78	6.38	
Vimentin	9.42	10.01	
CD44	8.85	5.61	
RPLO	11.27	10.16	Housekeeping gene

Supplementary Fig. 15. LEVs contain proteins and RNA. (**A**) BCA assay for estimation of protein concentration for MDA-MB 231 control cells, MDA-MB 231 cells post transit and their derived LEVs (n=4). (B) BSA standard curve (left) and respective data points for control cells, cells post transit and LEVs (right) (n=4). (C) Total RNA content (ng) measured from $2x10^5$ LEVs (n=1). (D) List of mRNA molecules that were present in purified LEVs, validated via reverse transcription polymerase chain reaction with their CT values against a housekeeping gene (RPLO) (n=2).

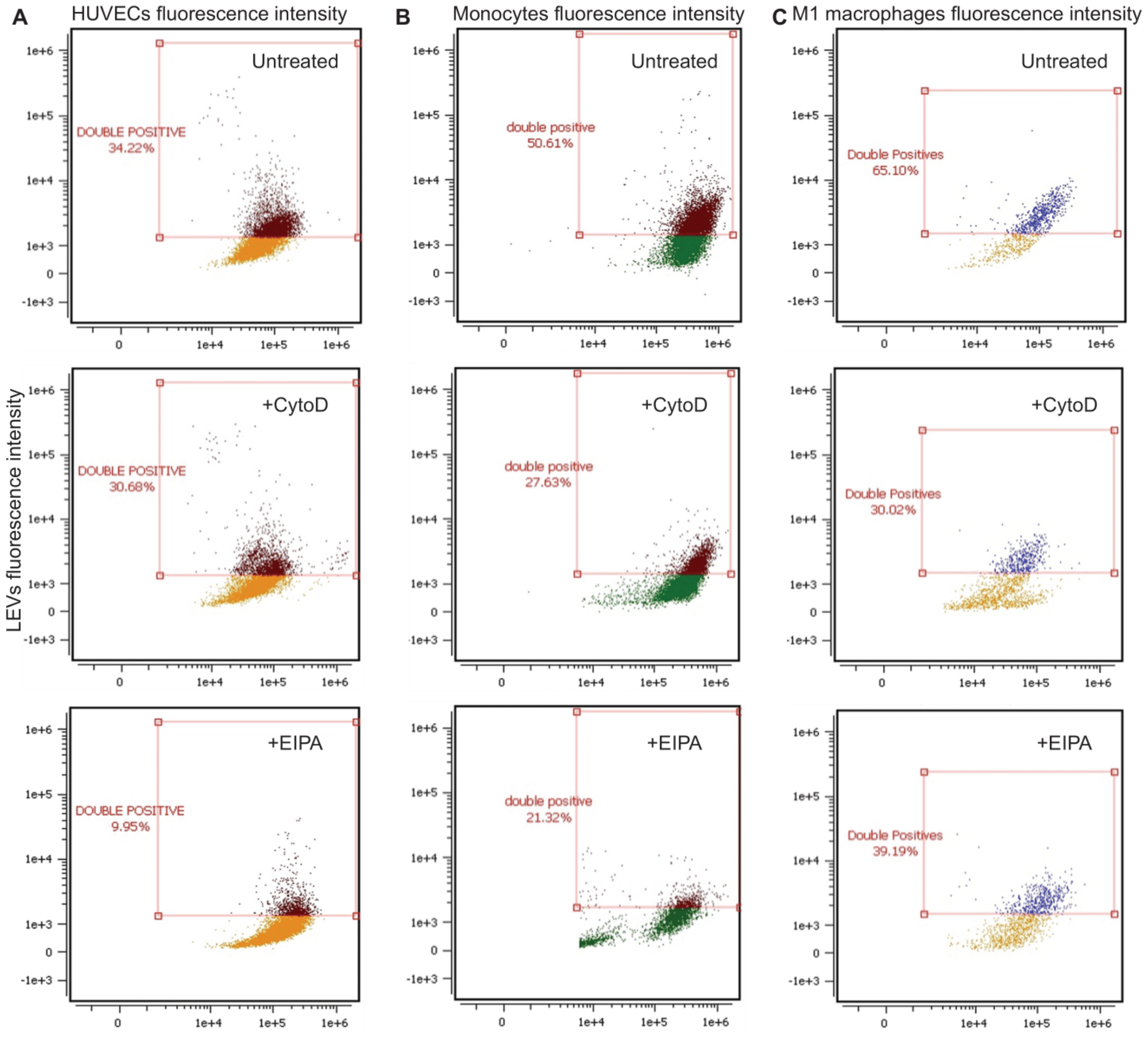

Supplementary Fig. 16. Internalization of tumor-cell derived LEVs by HUVECs, monocytes and M1 macrophages. (**A-C**) All data were obtained by flow cytometry. Dot plots of double positive events of cells (x axis) that have internalized LEVs (y axis) after 16 hr co-culture (top) and/or pre-treatment with Cyto-D (middle) or EIPA (bottom), for HUVECs (A), monocytes (B) and M1 macrophages (C). Cells were stained with CMTPX cell tracker (x axis fluorescence intensity) and LEVs were stained with CMFDA cell tracker (y axis fluorescence intensity).

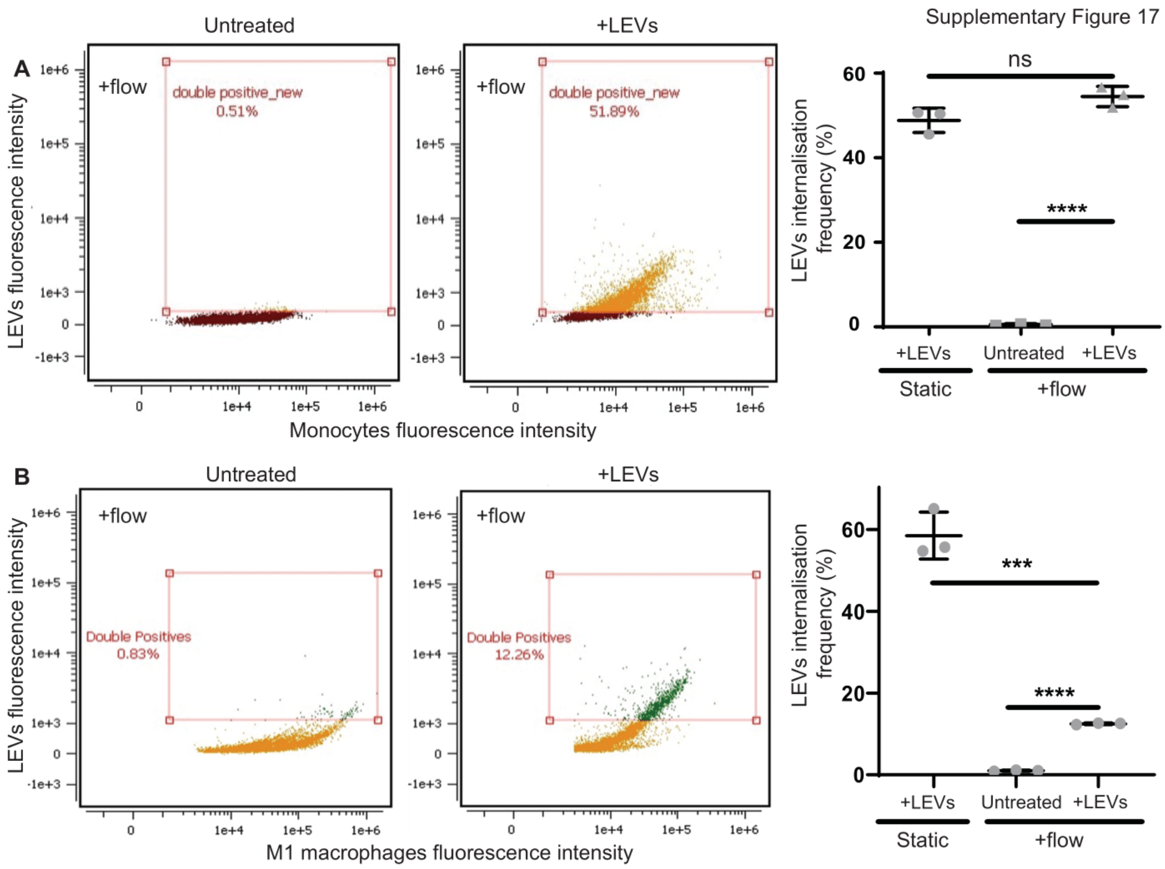

Supplementary Fig. 17. Internalization of tumor-cell derived LEVs by monocytes and M1 macrophages under flow conditions. (**A**) Dot plots of untreated (left) or LEV-treated monocytes (middle) & quantification (right) of internalized LEVs by monocytes under flow conditions (n=3). (B) Dot plots of untreated (left) or LEV-treated M1 macrophages (middle) & quantification (right) of internalized LEVs by M1 macrophages under flow conditions (n=3). Cells were stained with CMTPX cell tracker (x axis fluorescence intensity) and LEVs were stained with CMFDA cell tracker (y axis fluorescence intensity).

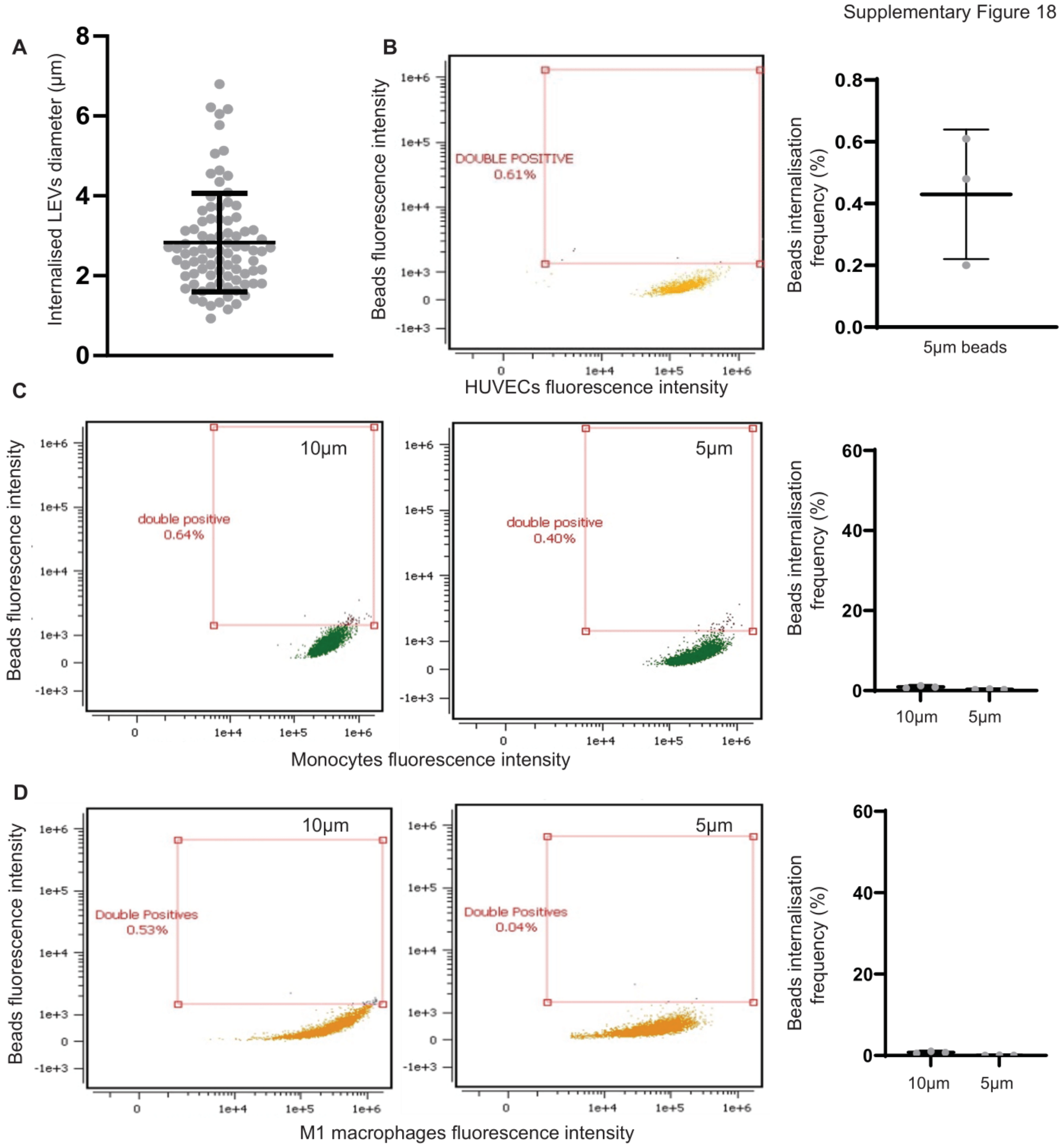

Supplementary Fig. 18. Investigation of fluorescent beads internalization by HUVECs, monocytes and M1 macrophages. (**A**) Diameter (µm) of internalized LEVs by HUVECs (n= 87 LEVs), verified by confocal microscopy. (B) Dot plot (left) & quantification (right) of 5 µm beads internalized by HUVECs (n=3). (C) Dot plots of 10 µm beads (left) and 5 µm beads (middle) & quantification (right) of their internalization by monocytes (n=3). (D) Dot plots of 10 µm beads (left) and 5 µm beads (middle) & quantification (right) of their internalization by M1 macrophages (n=3). All data excluding (A) were obtained from flow cytometry analysis. Cells were stained with CMTPX cell tracker (x axis fluorescence intensity) and fluorescent beads were purchased and used (y axis fluorescence intensity).

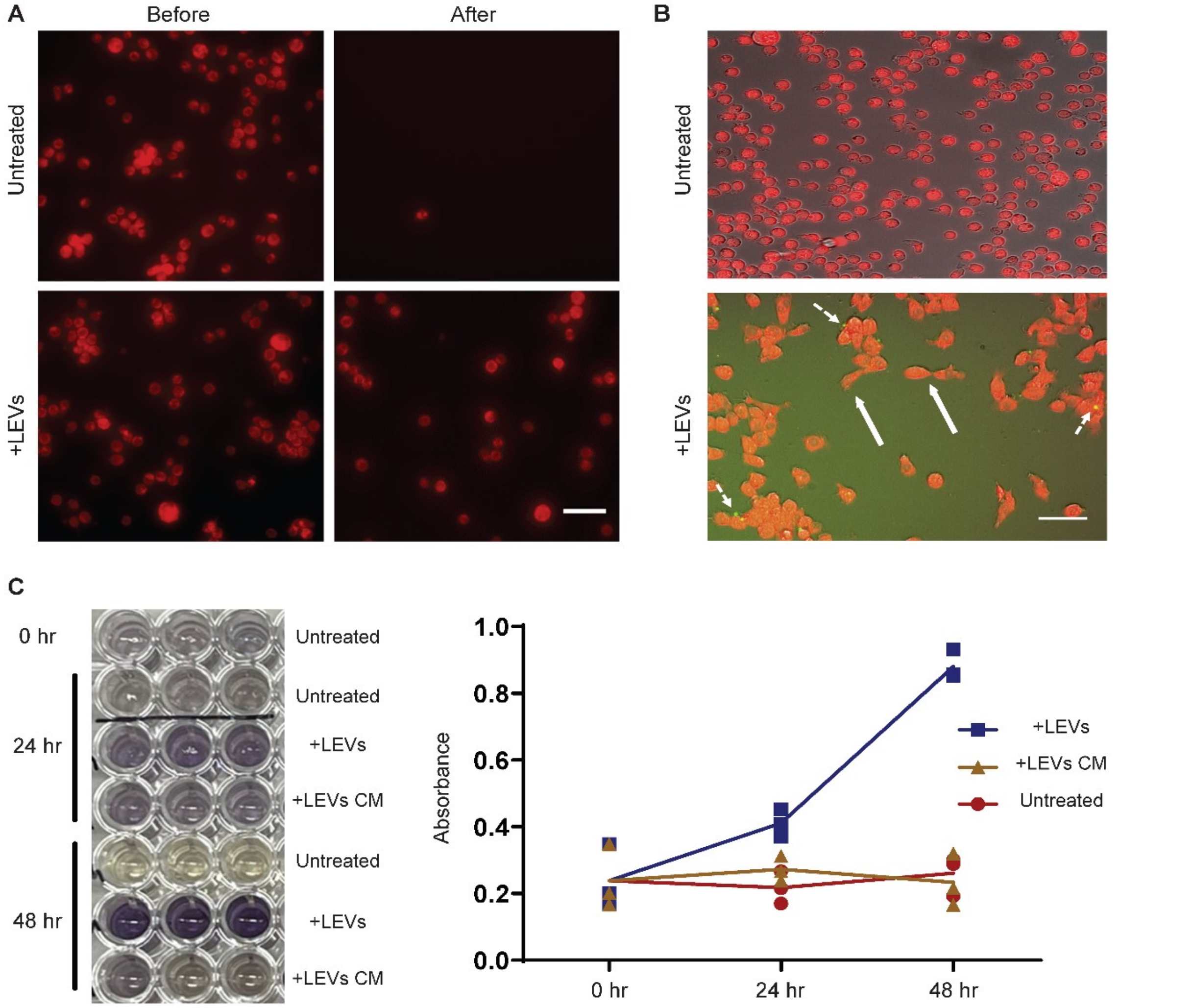

Supplementary Fig. 19. Impact of tumor cell-derived LEVs on monocytes. (**A**) Images of fluorescently tagged monocytes with CMTPX cell tracker (red) (untreated-top or LEV-treated-bottom) that remained adhered before (left) and after (right) cell media removal. Scale bar: 50 µm. (B) Images of fluorescently tagged untreated (top) or LEV treated monocytes (bottom), stained with CMTPX cell tracker (red) after 30hr co-culture. LEVs-were pre-stained with CMFDA cell tracker (green). Dashed arrows indicate locations that LEVs are in proximity to monocytes. Full arrows indicate monocytes that have assumed stretched morphologies. Scale bar: 50 µm. (C) Absorbance assay (left) & quantification (right) of proliferation rates at 0, 24 and 48 hr for untreated monocytes, monocytes that were treated with LEVs or monocytes treated with CM from LEVs (n=3).

Supplementary Figure 20

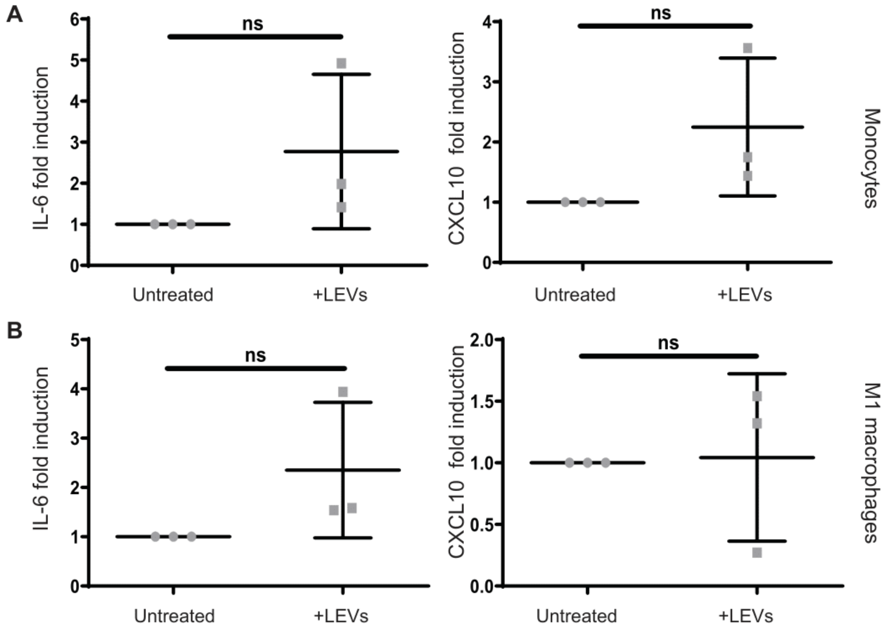

Supplementary Fig. 20. Investigation of tumor-cell derived LEVs on monocytes gene expression. (**A**) Reverse transcription polymerase chain reaction of untreated or LEV-treated monocytes (30hr) for IL-6 (left) and CXCL10 (right) genes (n=3). (B) Reverse transcription polymerase chain reaction of untreated or LEV-treated M1 macrophages (30hr) for IL-6 (left) and CXCL10 (right) genes (n=3).

Supplementary Figure 21

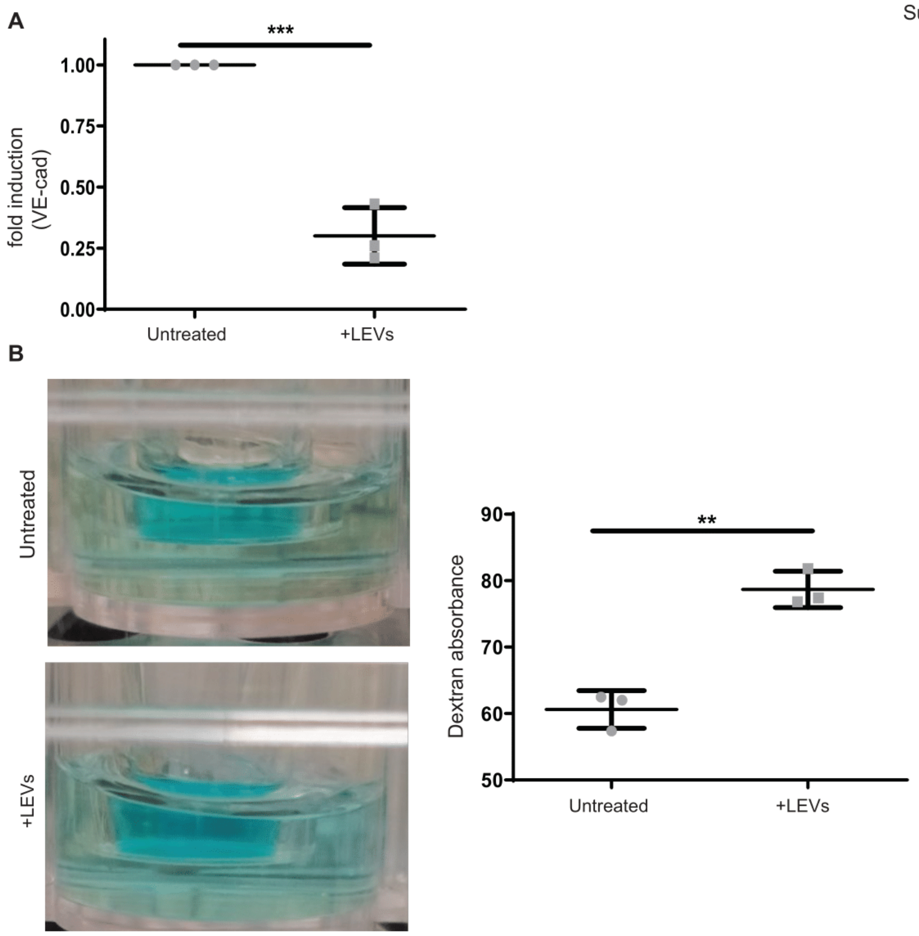

Supplementary Fig. 21. Tumor cell-derived LEVs reduce VE-cad mRNA on HUVECs and increase dextran permeability on HUVEC monolayers. (**A**) Reverse transcription polymerase chain reaction of untreated or LEV-treated HUVECs (30hr) for VE-cad gene (n=3). (B) Images (left) of untreated (top) or LEV-pre-treated (bottom) HUVEC monolayers grown in transwell inserts & quantification of absorbance (right) of permeated dextran, collected from the media (n=3).

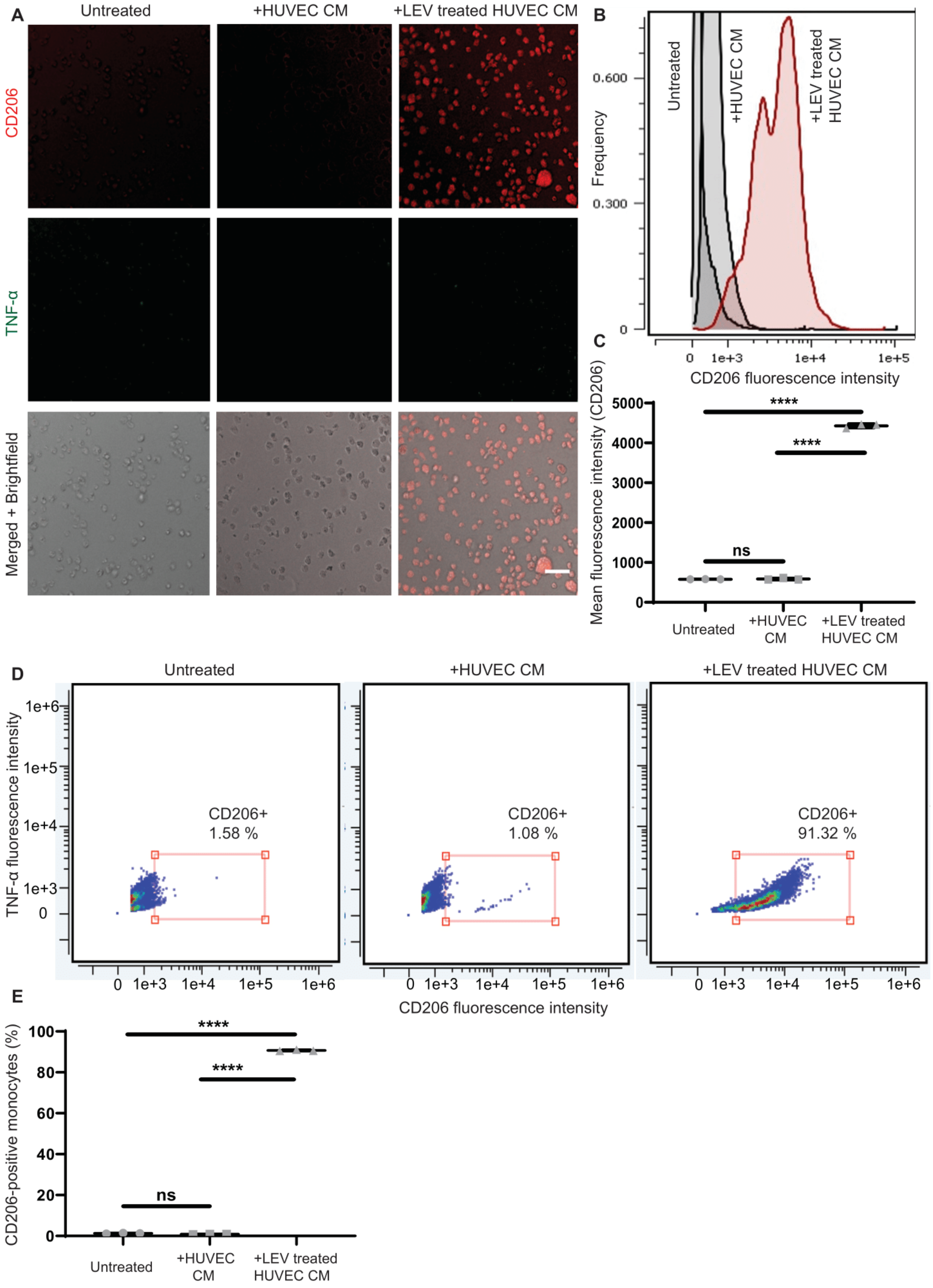
A
Untreated
+HUVEC CM
+LEV treated HUVEC CM
CD206
TNF-α
Merged + Brightfield
B
Frequency
0.600
0.300
0
Untreated
+HUVEC CM
+LEV treated HUVEC CM
0
1e+3
1e+4
1e+5
CD206 fluorescence intensity
C
Mean fluorescence intensity (CD206)
5000
4000
3000
2000
1000
0
****
****
ns
Untreated
+HUVEC CM
+LEV treated HUVEC CM
D
Untreated
+HUVEC CM
+LEV treated HUVEC CM
TNF-α fluorescence intensity
1e+6
1e+5
1e+4
1e+3
0
CD206+
1.58 %
CD206+
1.08 %
CD206+
91.32 %
0 1e+3 1e+4 1e+5 1e+6
CD206 fluorescence intensity
E
CD206-positive monocytes (%)
100
80
60
40
20
0
****
****
ns
Untreated
+HUVEC CM
+LEV treated HUVEC CM

Supplementary Fig. 22. CM from LEV-pre-treated HUVECs polarizes monocytes to M2 macrophages. (**A**) Multifluorescent images of untreated monocytes (left) or monocytes that were treated either with CM from untreated HUVECs (middle) or CM from LEV-pre-treated HUVECs (right). Monocytes were stained for CD206 (red) and TNF-α (green). Scale bar: 50 µm. (B-C) Histogram (B) & quantification (C) of CD206 mean fluorescence intensity for untreated monocytes, monocytes treated with CM from untreated HUVECs and monocytes treated with CM from LEV-pre-treated HUVECs, analyzed via flow cytometry (n=3). (D-E) Dot plots (D) & quantification (E) of CD206 positive events for untreated monocytes (left), monocytes treated with CM from untreated HUVECs (middle) and monocytes treated with CM from LEV-pre-treated HUVECs (right), analyzed via flow cytometry (n=3). CD206 and TNF-α fluorescence intensity are represented in x and y axes, respectively.

Supplementary Table 1.

Microfluidic capillary designs. Design parameters: a) d_0, d_1 and d_2 represent effective diameter of parent and two daughter bifurcation channels, b) θ° represents the angle between daughter bifurcation channels, c) Symmetry between two daughter channels represents equal angle deviation from the parent channel. Non-bifurcated devices represent a straight channel without branching. Non-constricted devices represent large channels that do not constrict cells.

**Name**	**d_0 (μm)**	**d_1 (μm)**	**d_2 (μm)**	**Angle (θ°)**	**Symmetry**
EWS	10	6.3	6.3	87°	YES
EWA	10	6.3	6.3	87°	NO
UWS	10	5.5	7	87°	YES
UWA	10	5.5	7	87°	NO
ENS	10	6.3	6.3	43.5°	YES
ENA	10	6.3	6.3	43.5°	NO
UNS	10	5.5	7	43.5°	YES
UNA	10	5.5	7	43.5°	NO
ENA_5/5	7.9	5	5	43.5°	NO
ENA_7/7	11.3	7	7	43.5°	NO
ENA_9/9	14.7	9	9	43.5°	NO
UNA_5/7	9.6	5	7	43.5°	NO
UNA_5/9	11.4	5	9	43.5°	NO
Non-bifurcated	7	N/A	N/A	N/A	N/A
Non-constricted	50	20	20	87°	YES

Supplementary Table 2.

Proteins identified in MDA-MB 231 cell-derived LEVs, via mass spectrometry based proteomic profiling, using LC-MS DIA analysis.

See attached excel file *Table S2*.

Supplementary Table 3.

MISEV Category 1 transmembrane proteins identified in MDA-MB 231 cell-derived LEVs, via mass spectrometry based proteomic profiling.

Transmembrane protein	MISEV Category 1
HLA-A	Non-tissue specific (1A)
ADAM10	Non-tissue specific (1A)
BSG	Non-tissue specific (1A)
CD47	Non-tissue specific (1A)
CD55	Non-tissue specific (1A)
CD59	Non-tissue specific (1A)
CD81	Non-tissue specific (1A)
CD82	Non-tissue specific (1A)
GNA11	Non-tissue specific (1A)
GNA13	Non-tissue specific (1A)
GNAI1	Non-tissue specific (1A)
GNAI2	Non-tissue specific (1A)
GNAI3	Non-tissue specific (1A)
GNAO1	Non-tissue specific (1A)
GNAQ	Non-tissue specific (1A)
ITGA1	Non-tissue specific (1A)
ITGA2	Non-tissue specific (1A)
ITGA3	Non-tissue specific (1A)
ITGA5	Non-tissue specific (1A)
ITGA6	Non-tissue specific (1A)
ITGB1	Non-tissue specific (1A)
ITGB3	Non-tissue specific (1A)
ITGB4	Non-tissue specific (1A)
ITGB5	Non-tissue specific (1A)
LAMP1	Non-tissue specific (1A)
LAMP2	Non-tissue specific (1A)
NT5E	Non-tissue specific (1A)
ABCC1	Cell/tissue specific (1B)
APP	Cell/tissue specific (1B)
CD9	Cell/tissue specific (1B)
EPCAM	Cell/tissue specific (1B)
ERBB2	Cell/tissue specific (1B)
HLA-DRA	Cell/tissue specific (1B)

Supplementary Table 4.

MISEV Category 2 cytosolic proteins identified in MDA-MB 231 cell-derived LEVs, via mass spectrometry based proteomic profiling.

Transmembrane protein	MISEV Category 2
ARF6	Proteins with lipid or protein binding ability (2A)
FLOT1	Proteins with lipid or protein binding ability (2A)
FLOT2	Proteins with lipid or protein binding ability (2A)
PDCD6IP	Proteins with lipid or protein binding ability (2A)
RHOA	Proteins with lipid or protein binding ability (2A)
SDCBP	Proteins with lipid or protein binding ability (2A)
TSG101	Proteins with lipid or protein binding ability (2A)
VPS4A	Proteins with lipid or protein binding ability (2A)
VPS4B	Proteins with lipid or protein binding ability (2A)
HSP90AA1	Proteins with lipid or protein binding ability (2A)
HSP90AB1	Proteins with lipid or protein binding ability (2A)
HSP90B1	Proteins with lipid or protein binding ability (2A)
HSPA13	Proteins with lipid or protein binding ability (2A)
HSPA14	Proteins with lipid or protein binding ability (2A)
HSPA4	Proteins with lipid or protein binding ability (2A)
HSPA4L	Proteins with lipid or protein binding ability (2A)
HSPA5	Proteins with lipid or protein binding ability (2A)
HSPA8	Proteins with lipid or protein binding ability (2A)
HSPA9	Proteins with lipid or protein binding ability (2A)
HSPB1	Proteins with lipid or protein binding ability (2A)
HSPB11	Proteins with lipid or protein binding ability (2A)
HSPBP1	Proteins with lipid or protein binding ability (2A)
HSPD1	Proteins with lipid or protein binding ability (2A)
HSPE1	Proteins with lipid or protein binding ability (2A)
HSPG2	Proteins with lipid or protein binding ability (2A)
HSPH1	Proteins with lipid or protein binding ability (2A)
GAPDH	Promiscuous incorporation in EVs (2B)
ACTBL2	Promiscuous incorporation in EVs (2B)
ACTL6A	Promiscuous incorporation in EVs (2B)
ACTN1	Promiscuous incorporation in EVs (2B)
ACTN4	Promiscuous incorporation in EVs (2B)
ACTR10	Promiscuous incorporation in EVs (2B)
ACTR1A	Promiscuous incorporation in EVs (2B)
ACTR1B	Promiscuous incorporation in EVs (2B)
ACTR2	Promiscuous incorporation in EVs (2B)
ACTR3	Promiscuous incorporation in EVs (2B)
TUBA1C	Promiscuous incorporation in EVs (2B)
TUBA4A	Promiscuous incorporation in EVs (2B)
TUBB	Promiscuous incorporation in EVs (2B)
TUBB1	Promiscuous incorporation in EVs (2B)
TUBB2A	Promiscuous incorporation in EVs (2B)
TUBB2B	Promiscuous incorporation in EVs (2B)
TUBB3	Promiscuous incorporation in EVs (2B)

TUBB4A	Promiscuous incorporation in EVs (2B)
TUBB4B	Promiscuous incorporation in EVs (2B)
TUBB6	Promiscuous incorporation in EVs (2B)
TUBG1	Promiscuous incorporation in EVs (2B)
TUBGCP2	Promiscuous incorporation in EVs (2B)
TUBGCP3	Promiscuous incorporation in EVs (2B)
TUBGCP5	Promiscuous incorporation in EVs (2B)
TUBGCP6	Promiscuous incorporation in EVs (2B)

Supplementary Table 5.

List of analyzed proteins via immunocytochemistry (including acronyms), on cells or LEVs, and primary/secondary antibodies used (with concentrations).

**Protein**	**Cell**	**Primary antibody (final concentration)**	**Secondary antibody (final concentration)**
Cytokeratin 18 (CK18)	MDA-MB 231 cells or LEVs from MDA-MB 231	Primary rabbit anti human monoclonal antibody CK18 (1 µg/ml)	Goat anti rabbit AlexaFluor 488 (2 µg/ml)
Cluster of differentiation (CD81)	MDA-MB 231 cells or LEVs from MDA-MB 231	Primary rabbit anti human monoclonal antibody CD81 (0.66 µg/ml)	Goat anti rabbit AlexaFluor 488 (2 µg/ml)
Heat shock protein 90 (HSP90)	MDA-MB 231 cells or LEVs from MDA-MB 231	Primary rabbit anti human monoclonal antibody HSP90 (1.74 µg/ml)	Goat anti rabbit AlexaFluor 488 (2 µg/ml)
Filamentous actin (F-actin)	MDA-MB 231 cells	Phalloidin 647 (1000 time dilution from stock vial-Abcam ab176759)	N/A
Vascular endothelial cadherin (VE-cad)	HUVECs	Primary rabbit anti human monoclonal antibody VE-cad (1 µg/ml)	Goat anti rabbit AlexaFluor 488 (2 µg/ml)
Vascular cell adhesion molecule (VCAM)	HUVECs	Primary rabbit anti human monoclonal antibody VCAM (2 µg/ml)	Goat anti rabbit AlexaFluor 488 (2 µg/ml)
Tumor necrosis factor α (TNF-α)	Monocytes	Primary rabbit anti human monoclonal antibody TNF-α (0.09 µg/ml)	Goat anti rabbit AlexaFluor 488 (2 µg/ml)

Cluster of differentiation (CD206)	Monocytes	Primary rabbit anti human monoclonal antibody PE/Cy7® Anti-Mannose Receptor (100 times dilution)	Goat anti rabbit AlexaFluor 647 (2 µg/ml)
C-X-C motif chemokine (CXCL10)	Monocytes	Primary rabbit anti human monoclonal antibody CXCL10 (2.5 µg/ml)	Goat anti rabbit AlexaFluor 488 (2 µg/ml)

Supplementary Table 6.

List of excitation and emission values used for dot plots generation via flow cytometry.

Type	x axis	y axis
MDA-MB 231 cells	Hoechst fluorescence intensity (Ex/Em: 405/456)	CK18 or CD81 or HSP90 fluorescence intensity (Ex/Em: 488/528)
LEVs	Red cell tracker fluorescence intensity (Ex/Em: 561/611)	CK18 or CD81 or HSP90 fluorescence intensity (Ex/Em: 488/528)
HUVECs	N/A	VE-cad fluorescence intensity (Ex/Em: 488/528)
Monocytes	CD206 fluorescence intensity (Ex/Em: 561/611)	TNF-a fluorescence intensity (Ex/Em: 488/528)

Supplementary Table 7.

List of mRNA molecules (with acronyms), including forward and reverse sequence (primers).

Gene name	Forward primer	Reverse primer
Interleukin 6 (IL-6)	GGTCCAGTTGCCTTCTCCCTG	TGCCCATGCTACATTTGCCG
C-X-C motif chemokine 10 (CXCL10)	TGAGCCTACAGCAGAGGAACC	GCCTCTGTGTGGTCCATCCTT
Fibronectin (FN)	GATGCACCATCCAACCTGCG	GATTGAGTCCCGGACCGTGT
Aldehyde dehydrogenase 1 (ALDH1)	CCCATCACAGGAGAGAAC	CCCTTAAATGCCATCCTG
B-cell lymphoma 2 (BCL2)	CAAGAAACTTCTACGACAGC	AAGCCATTTTCCTCTTCTTG
Cluster of differentiation (CD44)	TTATCAGGAGACCAAGACAC	ATCAGCCATTCTGGAATTTG
Vimentin (Vim)	GGAAACTAATCTGGATTCACTC	CATCTCTAGTTTCAACCGTC
Ribosomal protein large P (RPLO)	CGGTTTCTGATTGGCTAC	ACGATGTCACTTCCACG
Cluster of differentiation (CD206)	CGTTCGGTTCACCCACTGGA	ACATCCCATAAGCCCCCTGC
Arginase 1 (ARG-1)	TGGCAAGGTGGCAGAAGTCA	TGGCATGGCCAGAGATGCTT
Vascular endothelial cadherin (VE-cad)	GCCCACAGGCACGATCTGTT	CATCCGGTTCTGGGGCTCAT

Supplementary Movie 1. MDA-MB 231 cancer cell sheds LEVs in capillary bifurcation.

A MDA-MB 231 cell shedding LEVs in capillary bifurcation variant UNA. Cytoplasm was stained with CMFDA cell tracker (green) and nuclei with Hoechst-33342 (blue).

Supplementary Movie 2. CDX19 explant cell sheds a LEV in capillary bifurcation.

A CTC-derived CDX19 cell shedding in capillary bifurcation variant UNA_5/9 a single LEV. Cytoplasm was stained with CMFDA cell tracker (green).

Supplementary Movie 3. Patient CTC sheds a LEV in capillary bifurcation.

Patient CTC (isolated from small cell lung carcinoma patient) shedding in capillary bifurcation variant UNA_5/9 a single LEV. Cytoplasm was stained with CMFDA cell tracker (green) and nuclei with Hoechst-33342 (blue).

Supplementary Movie 4. Large cluster sheds multiple LEVs in capillary bifurcation.

A multicellular MDA-MB 231 cell cluster shedding in capillary bifurcation variant UNA_5/9 multiple LEVs. Cytoplasm was stained with CMFDA cell tracker (green) and nuclei with Hoechst-33342 (blue).

Supplementary Movie 5. Non-shedding cancer cell transits in a capillary bifurcation.

A non-shedding MDA-MB 231 cell transiting in capillary bifurcation variant ENA. Cytoplasm was stained with CMFDA cell tracker (green).

Supplementary Movie 6. Cancer cell undergoes lysis post-shedding in a capillary bifurcation.

A MDA-MB 231 cell shedding in capillary bifurcation variant EWA and undergoing lysis. Cytoplasm was stained with Calcein-AM (green) and nuclei with Hoechst-33342 (blue).

Supplementary Movie 7. Co-culture of endothelial cells with LEVs.

A human umbilical vein endothelial (HUVEC) cell near LEVs. HUVECs were stained with CMFDA cell tracker (green) and LEVs with CMTPX cell tracker (red).
